# The role of cardiovascular risk factors in maternal cardiovascular disease according to offspring birth characteristics in the HUNT study

**DOI:** 10.1038/s41598-021-99478-4

**Published:** 2021-11-26

**Authors:** Eirin B. Haug, Amanda R. Markovitz, Abigail Fraser, Håvard Dalen, Pål R. Romundstad, Bjørn O. Åsvold, Janet W. Rich-Edwards, Julie Horn

**Affiliations:** 1 Department of Public Health and Nursing, Faculty of Medicine and Health Sciences, K.G. Jebsen Center for Genetic Epidemiology, NTNU, Norwegian University of Science and Technology, Postboks 8905, 7491 Trondheim, Norway; 2 Department of Population Health Sciences, Bristol Medical School and MRC Integrative Epidemiology Unit at the University of Bristol, Bristol, UK; 3 Department of Epidemiology, Harvard T.H. Chan School of Public Health, Harvard University, Boston, MA USA; 4 Division of Women’s Health, Department of Medicine, Brigham and Women’s Hospital and Harvard Medical School, Boston, MA USA; 5 Mathematica, Cambridge, MA USA; 6 Department of Medicine, Levanger Hospital, Nord-Trøndelag Hospital Trust, Levanger, Norway; 7 Department of Circulation and Medical Imaging, NTNU, Norwegian University of Science and Technology, Trondheim, Norway; 8 Clinic of Cardiology, St. Olavs Hospital, Trondheim University Hospital, Trondheim, Norway; 9 Department of Public Health and Nursing, NTNU, Norwegian University of Science and Technology, Trondheim, Norway; 10 Department of Endocrinology, Clinic of Medicine, St. Olavs Hospital, Trondheim University Hospital, Trondheim, Norway; 11 Department of Obstetrics and Gynecology, Levanger Hospital, Nord-Trøndelag Hospital Trust, Levanger, Norway

**Keywords:** Cardiovascular diseases, Risk factors, Epidemiology

## Abstract

A history of preterm or small (SGA) or large (LGA) for gestational age offspring is associated with smoking and unfavorable levels of BMI, blood pressure, glucose and lipids. Whether and to what extent the excess cardiovascular risk observed in women with these pregnancy complications is explained by conventional cardiovascular risk factors (CVRFs) is not known. We examined the association between a history of SGA, LGA or preterm birth and cardiovascular disease among 23,284 parous women and quantified the contribution of individual CVRFs to the excess cardiovascular risk using an inverse odds weighting approach. The hazard ratios (HR) between SGA and LGA offspring and CVD were 1.30 (95% confidence interval (CI) 1.15, 1.48) and 0.89 (95% CI 0.76, 1.03), respectively. Smoking explained 49% and blood pressure may have explained ≈12% of the excess cardiovascular risk in women with SGA offspring. Women with preterm birth had a 24% increased risk of CVD (HR 1.24, 95% CI 1.06, 1.45), but we found no evidence for CVRFs explaining any of this excess cardiovascular risk. While smoking explains a substantial proportion of excess cardiovascular risk in women with SGA offspring and blood pressure may explain a small proportion in these women, we found no evidence that conventional CVRFs explain any of the excess cardiovascular risk in women with preterm birth.

## Introduction

Pregnancy complications are an early marker of later cardiovascular disease (CVD) in women and could provide an opportunity for targeted CVD prevention^1^. Both giving birth to small and large for gestational age (SGA and LGA) offspring have been reported to be associated with an increased risk of CVD^2–5^. For women experiencing preterm birth, the increased risk of CVD was according to a meta-analysis 63 percent^6^. Conventional cardiovascular risk factors such as body mass index (BMI), blood pressure, lipids, glucose and smoking are associated with offspring birthweight^7–12^ and preterm birth^9,13–15^. In order to inform targeted CVD prevention programs in women who gave birth to SGA or LGA offspring or experienced preterm birth, it is crucial to understand the relative contribution of these modifiable cardiovascular risk factors to the excess cardiovascular risk experienced by these women. Previously^16^, we examined the role of cardiovascular risk factors in explaining the excess cardiovascular risk in women who experienced preeclampsia or gestational hypertension, finding that a substantial part of the excess risk could be explained by elevated blood pressure and BMI. In this study, we take the same approach to investigate how much of the excess cardiovascular risk in women who experienced preterm birth or had SGA or LGA offspring can be explained by conventional cardiovascular risk factors.

## Methods

### Study population

The Trøndelag Health Study (HUNT) is a longitudinal population study that has invited all residents of Nord-Trøndelag county, Norway, to undergo extensive health assessments in the form of questionnaires, clinical measurements and blood samples^17^. So far, four surveys have been completed; HUNT1 (1984–1986), HUNT2 (1995–1997), HUNT3 (2006–2008) and HUNT4 (2017–2019). Participation rates for women were 89.9% in HUNT1, 75.5% in HUNT2 and 58.7% in HUNT3. The population in Nord-Trøndelag county is mostly White and considered to be representative of the population in Norway.

The Medical Birth Registry of Norway (MBRN) has recorded all births in Norway since 1967 together with demographic information and details on child and maternal health. We linked data from the HUNT study with the MBRN for 31,364 women who were found to have at least one recorded birth in the MBRN. Women (n = 4354) who gave birth to their first child after age 40 or turned 40 years after the 31st of December 2012, the end of the MBRN follow-up, were excluded as exposure status was defined as history of preterm birth, SGA or LGA offspring before age 40. We excluded births that were multiples or had gestational length < 22 or > 44 weeks, birth weight < 500 g, unlikely combinations of birth weight and gestational length (z-scores > 4 or < − 4) or lacked information on gestational length, birth weight or sex. Women who only had births fulfilling exclusion criteria were excluded from all analyses. For women who contributed with more than one birth, exposure status was based solely on births fulfilling inclusion criteria. This resulted in the exclusion of 666 women and 5138 births. For 1806 women we had incomplete information on covariates (described below) and these were also excluded. Additionally, we excluded 91 women with medical records of cardiovascular events before their start of follow-up, 193 women lacking date of cardiovascular event (self-reported in HUNT or reported in the MBRN) and 970 women who emigrated out of Nord-Trøndelag before the start of follow-up. A few women (n = 158) had a history of both SGA and LGA offspring and were for this reason excluded from the main analysis of this exposure. A total of 23,284 and 23,126 women were left for analysis of preterm birth and SGA/LGA offspring, respectively.

### Exposures

Z-scores of birth weight for sex and gestational age were calculated using Norwegian standards^18^. Gestational age at delivery used to be determined by the last menstrual period, but has since 1998 been based mainly on ultrasound examinations. SGA was defined as birth weight for gestational age and sex below the 10th percentile, and LGA as birth weight for gestational age and sex above the 90th percentile^18^. The remaining births were defined as appropriate for gestational age (AGA). Preterm birth was defined as births before 37 weeks gestation, but then also divided into moderate preterm (32–36 weeks) and very preterm (< 32 weeks) in a separate analysis. Exposure status was defined as history of SGA or LGA offspring or preterm birth before age 40.

### Covariates

We retrieved information about maternal birth year, mother’s age at birth and parity from the MBRN. From the HUNT survey questionnaires, measurements and interviews, we obtained information on maternal height, highest obtained educational level, work titles and family history of coronary heart disease in siblings or parents. We lacked information on educational level for 3461 women and instead used work titles to derive educational level based on recommendations from Statistics Norway^19^.

### Cardiovascular risk factors

Information about the cardiovascular risk factors BMI, systolic and diastolic blood pressure, non-fasting serum glucose, non-high-density lipoprotein (non-HDL) cholesterol and self-reported smoking status (never, former, current) was obtained from the most recent HUNT exam prior to the cardiovascular event or censoring. Details about the clinically measured cardiovascular risk factors have been reported previously^20^ and are included in the Supplementary Appendix S1.

### Cardiovascular events

We obtained information about hospital-diagnosed cardiovascular events from the medical records of patients with at least one record with an International Classifications of Diseases (ICD) 9 or ICD-10 code indicating CVD. Medical records were retrieved from the local hospitals serving Nord-Trøndelag county between September 1st 1987 and April 24th 2015. Two cardiologists reviewed all medical records according to established criteria (Supplementary Appendix S2) to confirm any valid cardiovascular diagnoses using ICD codes. We also retrieved information on cardiovascular deaths from first HUNT participation (January 16th 1984) until April 24th 2015 from the Norwegian Cause of Death Registry using ICD-9 and ICD-10 codes for the underlying cause of death (Table S1). For a visual depiction of the study timeline with data sources, see Supplementary Figure S1.

### Statistical analysis

We used Cox proportional hazards models with age as the time scale to estimate hazard ratios (HR) between SGA, LGA or preterm birth and cardiovascular events. Individual women were the unit of analysis and women entered our study either at September 1st 1987 (the start of CVD follow-up), their first HUNT exam or their 40th birthday, whichever came last. Follow-up lasted until either the first cardiovascular event, emigration out of Nord-Trøndelag county, death or April 24th 2015, whichever came first. To avoid guarantee time bias, we chose a conditional landmark analysis approach^21^ with age 40 as the landmark time point by which exposure was defined and CVD follow-up could start. The estimated HRs were adjusted for age (model 1), or age, maternal height and birth year, highest obtained educational level, parity before age 40 and family history of coronary heart disease in siblings or parents (model 2). To assess the proportional hazards assumption, we tested interactions between time (age) and individual covariates. For the analysis of the association between SGA or LGA and CVD the proportional hazards assumption was violated for the covariate indicating maternal birth year, and maternal birth year was included with an interaction with age.

In a separate analysis, we excluded women whose first birth was not recorded in the MBRN as these women could have been erroneously classified as unexposed. To allow inclusion of CVD events before age 40, we performed an analysis where exposure was assessed in first birth only and with follow-up starting from either first birth, first HUNT exam or September 1st 1987, whichever came last. In this analysis, we also adjusted for mother’s age at first birth. In the analysis of first birth, we excluded all women whose pregnancies fulfilled at least one of our exclusion criteria. In order to reduce the possibility that our results were driven by other pregnancy complications, we conducted a separate analysis excluding women with a history of hypertensive disorders of pregnancy, gestational diabetes, chronic diabetes mellitus and stillbirth. Since the validity of specific CVD subtypes is likely to be lower in the Cause of Death Registry compared to the validated hospital records, we also conducted a separate analysis using only validated CVD diagnoses. In two separate analyses, we examined if our results changed when also including women (n = 158) with a history of both SGA and LGA offspring in different pregnancies. To investigate if the association between SGA and CVD differed for a more severe form of SGA that may better reflect intrauterine growth retardation due to placental ischemic syndromes, we compared the risk of CVD in women with a history of severe SGA to that of women with history of AGA offspring. Severe SGA was in this analysis defined as offspring birth weight for gestational age and sex below the 5th percentile, and AGA was defined like previously as birth weight for gestational age and sex between the 10th and 90th percentile. Since history of preterm birth was not mutually exclusive for history of SGA or LGA offspring and vice versa, we also performed analyses where we excluded women with history of preterm birth and SGA and/or LGA offspring. Additionally, we assessed the association between CVD and combinations of SGA or LGA offspring and preterm birth by comparing the hazard of CVD between women with history of AGA offspring and women with history of either term or preterm LGA or SGA offspring.

Similar to the study by Tanz et al^22^ and our own work^16^, we have used a formal mediation analysis approach to estimate the proportion of excess cardiovascular risk in women who experienced preterm birth or had SGA offspring. Since LGA was not associated with an increased risk of CVD in our analysis, this exposure was not included as part of the mediation analysis. By using a stabilized inverse odds ratio (= inverse odds) weighting mediation analysis method^23,24^, we were able to decompose the association between our exposures and CVD into one natural direct effect of the exposures (pregnancy complications) on outcome and one natural indirect effect from exposures on outcome through mediators (cardiovascular risk factors)^25^. However, we do not conceptualize the cardiovascular risk factors as causal mediators as this requires a causal effect of exposure on mediators. Instead it is more likely that pre-pregnancy cardiovascular risk factors causally contribute to the development of pregnancy complications. In this study, the natural indirect effect is more appropriately interpreted as the part of the association between SGA or preterm birth and CVD that was explained by cardiovascular risk factors and the natural direct effect should be interpreted as the part that was not explained by these factors. We estimated the part of the association between SGA or preterm birth and CVD that was explained by BMI, blood pressure, non-fasting serum glucose, non-HDL cholesterol and smoking (indirect effect) and the part that was not explained by these factors (direct effect). These associations were adjusted for the same covariates as described above in model 2, but were in the case of the continuous cardiovascular risk factors BMI, blood pressure, serum glucose and non-HDL additionally adjusted for age of cardiovascular risk factors ascertainment. In our main analysis, the cardiovascular risk factors for 5362 (30%) women were measured or recorded before the end of their reproductive follow-up (age 40). However, the timing of measurement or collection of information about cardiovascular risk factors was less important as we do not assume that SGA or preterm birth substantially influence these cardiovascular risk factors. In a sensitivity analysis, we excluded women who had their cardiovascular risk factors measured or recorded before age 40. To investigate whether the proportion excess cardiovascular risk in women with history of SGA or preterm birth that was explained by cardiovascular risk factors differed by age of risk factor ascertainment, we performed another sensitivity analysis where we retrieved information about cardiovascular risk factors from the first HUNT exam (as opposed to the most recent exam). Lastly, we also examined the contributions of individual cardiovascular risk factors to the excess cardiovascular risk in women with history of severe SGA offspring, and in women with history of SGA offspring but without a history of hypertensive disorders of pregnancy, gestational diabetes, chronic diabetes mellitus and stillbirth. For a graphic illustration of the mediation analysis see Figure S2. All statistical analysis was performed with Stata 16.

### Ethical approval

This study was approved by the Regional Committee for Medical and Health Research Ethics on 10 May 2013 (Reference number: 2013/647/REK midt). Participants provided informed consent and all procedures performed in studies involving human participants were in accordance with the ethical standards of the institutional and/or national research committee.

## Results

In our study population, 4203 (18%) women had a history of SGA offspring, 3814 (16%) gave birth to at least one LGA offspring and 2549 (11%) women experienced at least one preterm birth (Table 1). Descriptive statistics of the included pregnancies can be found in Table S2. The cardiovascular risk factors were measured (BMI, blood pressure, glucose and non-HDL cholesterol) or collected (smoking information) at a median age of 50 years. During a median follow-up of 18 years, 1604 women experienced a cardiovascular event, of which 1494 (93%) were validated. Please find additional information about the timing of ascertainment of these cardiovascular risk factors in Supplementary Tables S3 and S4. A total of 205 women had a fatal CVD event, 530 experienced a myocardial infarction, 224 experienced heart failure and 834 had cerebrovascular disease.

**Table 1:** Maternal descriptive characteristics according to history of pregnancy complication.

	Fetal growth			Gestational length	
	History of SGA offspring (n = 4203)	History of AGA offspring (n = 15,109)	History of LGA offspring (n = 3814)	History of preterm birth (< 37 weeks) (n = 2109)	History of term birth (n = 21,175)
Birthyear, median (IQR)	1954 (1947–1961)	1954 (1946–1962)	1956 (1947–1964)	1955 (1948–1963)	1954 (1946–1962)
Age at 1st birth, median (IQR)	23 (20–26)	24 (21–28)	24 (21–27)	23 (20–27)	24 (21–27)
No. of included pregnancies (%)					
One	779 (19)	4691 (31)	550 (14)	320 (15)	5700 (27)
Two	1834 (44)	6557 (43)	1508 (40)	819 (39)	9123 (43)
Three or more	1590 (38)	3861 (26)	1756 (46)	970 (46)	6352 (30)
First birth recorded in the MBRN (%)					
Yes	3708 (88)	12,039 (80)	3208 (84)	1855 (88)	17,250 (81)
Family history of CVD (%)					
Yes	1640 (39)	5349 (35)	1300 (34)	732 (35)	7612 (36)
Education (%)				732 (35)	7612 (36)
Lower Secondary	1144 (27)	3792 (25)	872 (23)	546 (26)	5301 (25)
Upper secondary	1915 (46)	6529 (43)	1596 (42)	974 (46)	9140 (43)
Tertiary	1144 (27)	4788 (32)	1346 (35)	589 (28)	6734 (32)
Cardiovascular risk factors^a^					
BMI, mean (SD)	25.88 (4.56)	26.59 (4.74)	27.73 (4.98)	26.47 (4.82)	26.67 (4.78)
Systolic blood pressure, mean (SD)	128.60 (18.30)	127.71 (18.60)	126.94 (18.36)	127.65 (18.14)	127.76 (18.54)
Diastolic blood pressure, mean (SD)	74.51 (11.19)	73.61 (11.17)	73.08 (10.96)	74.16 (11.28)	73.65 (11.13)
Non-fasting serum glucose, mean (SD)	5.38 (1.17)	5.38 (1.24)	5.50 (1.57)	5.45 (1.53)	5.39 (1.26)
Non-HDL cholesterol, mean (SD)	4.24 (1.12)	4.20 (1.12)	4.20 (1.14)	4.24 (1.13)	4.21 (1.12)
Smoking status (%)					
Never	1225 (29)	5944 (39)	1837 (48)	777 (37)	8283 (39)
Former at last HUNT exam	1379 (33)	5158 (34)	1306 (34)	688 (33)	7214 (34)
Current at last HUNT exam	1599 (38)	4007 (27)	671 (18)	644 (31)	5678 (27)
Number of women smoking before index pregnancy (%)	2630 (63)	7723 (51)	1619 (42)	1127 (53)	10,928 (52)
Age of smoking initiation^b^, median (IQR)	17 (16–19)	18 (16–20)	17 (16–20)	17 (16–19)	17 (16–20)
Ever used antihypertensives	730 (17)	2351 (16)	586 (15)	423 (17)	3275 (16)
History of preterm birth	573 (14)	1043 (7)	434 (11)	N/A	N/A
History of SGA or LGA offspring	N/A	N/A	N/A	1066 (51)	7109 (34)

^a^Due to differential availability of cardiovascular risk factor measurements from the different HUNT exams and missing records, the number of women given at the top of the table are not exactly the same group as the number in which the cardiovascular risk factor summary statistics have been calculated.

^b^Calculated among smokers. *SGA *small for gestational defined as birth weight for gestational age and sex below the 10th percentile, *AGA* appropriate for gestational age, *LGA* large for gestational defined as birth weight for gestational age and sex above the 90th percentile, *IQR* interquartile range, *MBRN* Medical Birth Registry of Norway, *CVD* cardiovascular disease, *BMI* body mass index, *HDL* high-density lipoprotein, *SD* standard deviation, *N/A* not applicable.

Results from age-adjusted and multivariable adjusted models for the association between SGA, LGA or preterm birth and CVD are presented in Tables 2 and 3, and results from the multivariable adjusted model are discussed in the text below.

**Table 2:** Hazard ratios for cardiovascular events in women by history of SGA or LGA offspring.

	No. of women	Person-years	No. of events	Model 1^a^		Model 2^b^	
				Hazard ratio (95% CI)	*p*-value	Hazard ratio (95% CI)	*p*-value
**Any CVD**							
SGA	4203	72,353	329	1.31 (1.15, 1.48)	< 0.001	1.30 (1.15, 1.48)	< 0.001
AGA	15,109	260,172	1068	Ref		Ref	
LGA	3814	63,234	207	0.86 (0.74, 0.99)	0.042	0.89 (0.76, 1.03)	0.129
**Fatal CVD**							
SGA	4203	74,639	48	1.67 (1.20, 2.33)	0.002	1.86 (1.32, 2.61)	< 0.001
AGA	15,109	267,087	135	Ref		Ref	
LGA	3814	64,419	22	0.75 (0.48, 1.18)	0.222	0.81 (0.51, 1.28)	0.371
**Myocardial infarction**							
SGA	4203	73,713	117	1.42 (1.15, 1.75)	0.001	1.40 (1.13, 1.73)	0.002
AGA	15,109	264,698	348	Ref		Ref	
LGA	3814	64,059	65	0.83 (0.64, 1.08)	0.173	0.90 (0.69, 1.17)	0.431
**Heart failure**							
SGA	4203	74,433	42	1.40 (0.99, 1.98)	0.056	1.44 (1.02, 2.05)	0.041
AGA	15,109	266,402	144	Ref		Ref	
LGA	3814	64,240	38	1.23 (0.86, 1.76)	0.252	1.24 (0.86, 1.79)	0.239
**Cerebrovascular disease**							
SGA	4203	73,507	173	1.30 (1.09, 1.54)	0.003	1.28 (1.07, 1.52)	0.006
AGA	15,109	2,263,363	558	Ref		Ref	
LGA	3814	63,773	103	0.82 (0.66, 1.01)	0.064	0.85 (0.69, 1.06)	0.148

^a^Adjusted for age.

^b^Adjusted for age, highest obtained educational level, parity before age 40, maternal birth year, maternal height and family history of coronary heart disease. *SGA* small for gestational age, *LGA* large for gestational age, *CI* confidence interval, *CVD* cardiovascular disease, *AGA* appropriate for gestational age.

**Table 3:** Hazard ratios for cardiovascular events in women by history of preterm birth.

	No. of women	Person-years	No. of events	Model 1^a^		Model 2^b^	
				Hazard ratio (95% CI)	*p*-value	Hazard ratio (95% CI)	*p*-value
**Any CVD**							
Term	21,175	363,529	1464	Ref		Ref	
Preterm (< 37 weeks)	2109	34,906	150	1.23 (1.04, 1.45)	0.018	1.25 (1.06, 1.49)	0.009
**Fatal CVD**							
Term	21,175	373,086	185	Ref		Ref	
Preterm (< 37 weeks)	2109	35,792	21	1.50 (0.96, 2.36)	0.078	1.65 (1.04, 2.60)	0.033
**Myocardial infarction**							
Term	21,175	369,670	486	Ref		Ref	
Preterm (< 37 weeks)	2109	35,528	47	1.15 (0.85, 1.55)	0.362	1.20 (0.89, 1.62)	0.239
**Heart failure**							
Term	21,175	372,086	210	Ref		Ref	
Preterm (< 37 weeks)	2109	35,720	15	0.94 (0.56, 1.60)	0.829	0.96 (0.57, 1.63)	0.881
**Cerebrovascular disease**							
Term	21,175	368,066	763	Ref		Ref	
Preterm (< 37 weeks)	2109	35,274	75	1.18 (0.93, 1.50)	0.172	1.19 (0.94, 1.52)	0.148

^a^Adjusted for age.

^b^Adjusted for age, highest obtained educational level, parity before age 40, maternal birth year, maternal height and family history of coronary heart disease. *CI* confidence interval, *CVD* cardiovascular disease.

### SGA and LGA offspring

Women with a history of SGA offspring had a moderately increased risk of CVD (HR 1.30, 95% CI 1.15, 1.48) and of fatal CVD, myocardial infarction, heart failure and cerebrovascular disease (Table 2). Women with a history of LGA offspring had a reduced risk of CVD compared to women with AGA offspring, except for heart failure where there was a suggestion of possible increased risk. However, limited statistical power gave imprecise results for women with LGA offspring.

Restricting our analysis to women whose first birth was included in the MBRN attenuated the associations between SGA and CVD (Supplementary Table S5) and left those between LGA and CVD unchanged. In an analysis including only first births, estimates for the association between SGA or LGA and CVD were reduced further (Supplementary Table S6), but there was still indication of increased risk of CVD for mothers of SGA offspring. Excluding women with history of other pregnancy complications or restricting the analysis to validated cardiovascular events did not noticeably change the results (Supplementary Tables S7 and S8). The inclusion of women with a history of both SGA and LGA offspring, whether they were classified as SGA or LGA, also left our results unchanged. Compared to women with history of AGA offspring, women with a history of severe SGA had similar but slightly higher hazard ratios for CVD than women with history of SGA (Supplementary Table S9). Excluding women with history of preterm birth did not meaningfully change the association between history of SGA or LGA offspring and CVD (Supplementary Table 10). The combination of history of preterm birth and SGA offspring was associated with higher hazard ratios for most CVD outcomes compared to history of term births and SGA offspring (Supplementary Table S11). For women with history of LGA offspring the combination with history of preterm birth reversed the association with CVD (Supplementary Table 12).

Using the mediation analysis approach, we estimated that smoking explained 49% and that increased systolic and diastolic blood pressure may have explained ≈12% of the excess cardiovascular risk in women with history of SGA offspring, though lack of statistical significance prevented conclusive inference for blood pressure. (Table 4). The cardiovascular risk factors glucose, non-HDL cholesterol and BMI did not seem to explain any of the excess cardiovascular risk in women with history of SGA offspring. Results were similar when restricting this analysis to women who had their cardiovascular risk factors measured or recorded after age 40 (Supplementary Table S13). Excluding women with a history of hypertensive disorders of pregnancy, gestational diabetes, chronic diabetes mellitus and stillbirth resulted in a small decrease in the proportion excess cardiovascular risk accounted for by systolic and diastolic blood pressure and a small increase in the proportion accounted for by smoking (Supplementary Table S14). Defining severe SGA by birth weight for gestational age and sex below the 5th percentile, resulted in mostly similar but slightly higher estimates for the proportion excess cardiovascular risk that was accounted for by systolic and diastolic blood pressure (Supplementary Table S15). Results remained similar, but somewhat reduced (Supplementary Table S16) when using measurements or information about cardiovascular risk factors obtained from women’s first HUNT exam.

**Table 4:** Decomposition of the association between history of SGA offspring and cardiovascular disease into direct and indirect effects.

Cardiovascular risk factor	No. of women	Total effect^a^		Direct effect^b^		Indirect effect^c^		Proportion explained^d^
		Hazard ratio (95% CI)	*p*-value	Hazard ratio (95% CI)	*p*-value	Hazard ratio (95% CI)	*p*-value	Percentage
BMI^e^	19,033	1.24 (1.09, 1.41)	0.001	1.25 (1.09, 1.44)	0.002	0.99 (0.94, 1.05)	0.757	− 4
Systolic blood pressure^e^	19,021	1.24 (1.08, 1.42)	0.002	1.21 (1.04, 1.39)	0.011	1.03 (0.98, 1.08)	0.304	12
Diastolic blood pressure^e^	19,021	1.24 (1.08, 1.42)	0.002	1.21 (1.04, 1.40)	0.012	1.02 (0.97, 1.08)	0.401	11
Serum glucose^e^	17,683	1.25 (1.09, 1.43)	0.002	1.25 (1.08, 1.45)	0.004	1.00 (0.94, 1.05)	0.899	− 2
Serum non-HDL cholesterol^e^	17,388	1.23 (1.07, 1.42)	0.004	1.23 (1.06, 1.44)	0.008	1.00 (0.94, 1.06)	0.989	0
Smoking^f^	18,905	1.29 (1.13, 1.47)	< 0.001	1.14 (0.99, 1.31)	0.075	1.13 (1.07, 1.20)	< 0.001	49

^a^Association between history of SGA offspring and CVD compared to women with history of appropriate for age offspring. Hazard ratio differs from that in Table 2 due to additional adjustment for age of mediator ascertainment.

^b^Part of the association between history of SGA offspring and CVD that is not explained by the cardiovascular risk factor.

^c^Part of the association between history of SGA offspring and CVD that is explained by the cardiovascular risk factor.

^d^Proportion excess cardiovascular risk in women with history of SGA offspring that is explained by cardiovascular risk factor.

^e^Estimates are adjusted for age (used as time scale in the Cox proportional hazards model), age at measurement of the cardiovascular risk factor, highest obtained educational level, age at first birth, parity, first birth in the Medical Birth Registry of Norway, maternal height and birth year.

^f^Estimates are adjusted for age (used as time scale in the Cox proportional hazards model), highest obtained educational level, age at first birth, parity, first birth in the Medical Birth Registry of Norway, maternal height and birth year. *SGA* small for gestational age, *CVD* cardiovascular disease, *CI* confidence interval, *BMI* body mass index, *HDL* high-density lipoprotein.

### Preterm birth

Women who experienced preterm birth had a moderately increased risk of any CVD compared to women who had term births (HR 1.25, 95% CI 1.06, 1.49) (Table 3). Risk estimates were similar for moderate preterm birth (32–36 weeks), but inconclusive for the very preterm birth group (< 32 weeks) due to few events (Supplementary Table S17). Restricting the analysis to women whose first birth was recorded in the MBRN (Supplementary Table S18) or to first births (Supplementary Table S19) also gave similar results. Excluding women with history of other pregnancy complications or restricting the analysis to validated cardiovascular events did not alter our results either (Supplementary Tables S20 and S21). When excluding women with history of SGA or LGA offspring, the association between history of preterm birth and CVD remained of comparable magnitude (Supplementary Table 22).

All hazard ratios for indirect effects of cardiovascular risk factors on excess cardiovascular risk in women with history of preterm birth were not different than one, indicating that these risk factors did not account for any of the excess cardiovascular risk in these women, though insufficient statistical power prevented conclusive inferences (Table 5). Limiting the study population to women who had their cardiovascular risk factors measured or recorded after age 40 did not substantially alter the results (Supplementary Table S23). In the analysis where measurements of BMI, blood pressure, glucose, non-HDL cholesterol and information on smoking were retrieved from women’s first HUNT exam, results were also similar (Supplementary Table S24).

**Table 5:** Decomposition of the association between history of preterm birth and cardiovascular disease into direct and indirect effects.

Cardiovascular risk factor	No. of women	Total effect^a^		Direct effect^b^		Indirect effect^c^		Proportion explained^d^
		Hazard ratio (95% CI)	*p*-value	Hazard ratio (95% CI)	*p*-value	Hazard ratio (95% CI)	*p*-value	Percentage
BMI^e^	22,945	1.19 (0.99, 1.43)	0.070	1.23 (1.00, 1.50)	0.045	0.97 (0.90, 1.05)	0.429	− 18
Systolic blood pressure^e^	22,932	1.25 (1.05, 1.49)	0.011	1.26 (1.04, 1.52)	0.016	1.00 (0.92, 1.08)	0.930	− 2
Diastolic blood pressure^e^	22,933	1.18 (0.99, 1.41)	0.063	1.19 (0.98, 1.45)	0.075	0.99 (0.92, 1.08)	0.871	− 4
Serum glucose^e^	21,343	1.14 (0.94, 1.38)	0.192	1.16 (0.95, 1.43)	0.146	0.98 (0.90, 1.06)	0.560	− 19
Serum non-HDL cholesterol^e^	21,005	1.14 (0.93, 1.39)	0.208	1.18 (0.95, 1.47)	0.126	0.96 (0.88, 1.05)	0.370	− 30
Smoking^f^	22,789	1.27 (1.07, 1.51)	0.007	1.26 (1.05, 1.51)	0.015	1.01 (0.93, 1.09)	0.821	4

^a^Association between history of preterm birth and CVD compared to women with history of term birth. Hazard ratio differs from that in Table 2 due to additional adjustment for age of mediator ascertainment.

^b^Part of the association between history of preterm birth and CVD that is not explained by the cardiovascular risk factor.

^c^Part of the association between history of preterm birth and CVD that is explained by the cardiovascular risk factor.

^d^Proportion excess cardiovascular risk in women with history of preterm birth that is explained by cardiovascular risk factor.

^e^Estimates are adjusted for age (used as time scale in the Cox proportional hazards model), age at measurement of the cardiovascular risk factor, highest obtained educational level, age at first birth, parity, first birth in the Medical Birth Registry of Norway, maternal height and birth year.

^f^Estimates are adjusted for age (used as time scale in the Cox proportional hazards model), highest obtained educational level, age at first birth, parity, first birth in the Medical Birth Registry of Norway, maternal height and birth year. *CVD* cardiovascular disease, *CI* confidence interval, *BMI* body mass index, *HDL* high-density lipoprotein.

## Discussion

Women with a history of SGA offspring had a 30% increased risk of CVD later in life. We found no increased risk of CVD in women with LGA offspring; instead the estimates indicated a lower risk in this group of women. Using a novel mediation analysis approach, we found that approximately ½ of the excess cardiovascular risk in women who had SGA offspring could be explained by smoking and that a small proportion may be explained by elevated blood pressure. In our population, a history of preterm birth was associated with a 24% increased risk of CVD, but we could not find evidence for any of this excess risk being explained by conventional cardiovascular risk factors. Our observed association between history of SGA offspring and CVD mortality was generally consistent with the results from four larger Norwegian prospective cohort studies^2–4,26^. In contrast to our results, Morken et al.^2^ found that mothers of LGA offspring also had an increased risk of cardiovascular mortality. However, further analyses by Morken et al. revealed this only applied to the LGA offspring that were born preterm. Other comparable Scandinavian^27–29^ and international^5,6^ studies also found similar results to ours. To our knowledge, no other study has attempted to evaluate the role of individual cardiovascular risk factors in explaining the excess risk of CVD in women with a history of SGA offspring using a mediation analysis approach. Consistent with our results, Shaikh et al.^3,4^ reported reductions in the effect estimates when adjusting the association between SGA and CVD for cardiovascular risk factors. In one of their studies^3^, Shaikh et al. investigated the effect of adjusting the association between SGA and CVD for individual cardiovascular risk factors and found that adjusting for smoking attenuated the association by 40% and that adjusting for triglycerides, blood pressure and diabetes resulted in smaller reductions in the effect estimate. Our findings are also consistent with the life course trajectories of cardiovascular risk factors that we previously^12^ drew, where we observed that systolic and diastolic blood pressure were slightly elevated in women with history of SGA offspring compared to in women with AGA offspring.

Several studies^5,6^, including one Norwegian^30^, of the association between preterm first birth and CVD found comparable results to ours. A study by Tanz et al.^22^ of 70,182 women from the American Nurses’ Health Study II reported that 14.5% of the excess cardiovascular risk in women with a history of preterm birth was accounted for by hypertension, hypercholesterolemia, type 2 diabetes mellitus and BMI. Although we did not find evidence for cardiovascular risk factors accounting for any of the excess cardiovascular risk in women with history of preterm birth, it is possible that this was due to insufficient statistical power or that the cardiovascular risk factors in our study were continuously measured while those of Tanz et al.^22^ were dichotomous. Our results are, however, consistent with our previous findings that life course trajectories of cardiovascular risk factors in women with a history of normotensive preterm birth do not differ from those of women with history of normotensive term birth^31^.

In contrast to most other studies that purely relied on registry data and only collected information on fatal cardiovascular endpoints, our study included both fatal and non-fatal events retrieved from hospital records and the Cause of Death Registry, 93% of which were validated. The MBRN provided precise information about our exposures as was observed by Moth et al.^32^ who reported a positive predictive value of 95% for preterm birth and a 100% positive predictive value for low or high birthweight. By linking to the HUNT study, we were in the rare position of being able to examine the role of smoking and precisely measured cardiovascular risk factors in CVD using a novel and formal mediation analysis, while also adjusting for relevant confounders. Our results are probably generalizable to populations that, like Norway, have free access to good health care and are subject to the same degree of clinical follow-up. Secular changes in cardiovascular risk factors could potentially alter how much of the excess cardiovascular risk is explained by individual cardiovascular risk factors. During our study period, mean age-adjusted BMI increased^33^ while the prevalence of smoking decreased noticeably from the late 1990s^34^. Future studies may find a diminishing role for smoking and different contributions from other cardiovascular risk factors to excess cardiovascular risk in women with history of preterm birth or SGA offspring compared to our study. Since we also included some women with a first birth that was not recorded in the MBRN, some women may have been misclassified as not having a history of pregnancy complications in the main analysis. However, restricting the analysis to those with their first births recorded in the MRBN did not noticeably change our results. Furthermore, additional analyses indicated that the associations between CVD and our exposures were not driven by other pregnancy complications. Starting follow-up at age 40 may have caused the exclusion of early cardiovascular events but starting follow-up earlier did not change our results.

## Conclusion

We have shown that the conventional and modifiable cardiovascular risk factor smoking explains a substantial proportion of the excess cardiovascular risk in women with a history of SGA offspring and that blood pressure may explain a smaller proportion. Our results do not indicate that conventional cardiovascular risk factors explain any of the excess cardiovascular risk in women with a history of preterm birth. Based on our results, women who gave birth to SGA offspring may benefit from early prevention programs that encourage smoking cessation and possibly also prevention programs that aim to reduce their blood pressure. Further research is needed to develop such programs and quantify their effects on the excess cardiovascular risk in these women.

## Supplementary Information

Supplementary Information.

## Abbreviations

AGA: Appropriate for gestational age
BMI: Body mass index
CVRF: Cardiovascular risk factor
CVD: Cardiovascular disease
HDL: High-density lipoprotein
HR: Hazard ratio
ICD: International Classifications of Diseases
LGA: Large for gestational age
MBRN: Medical Birth Registry of Norway
SGA: Small for gestational age
The HUNT study: Trøndelag Health study
