# Supplementary material for: The role of cardiovascular risk factors in maternal cardiovascular disease according to offspring birth characteristics in the HUNT study
Source: Sci Rep. 2021 Nov 26;11:22981. doi: 10.1038/s41598-021-99478-4 (PMC8626478; doi:10.1038/s41598-021-99478-4)
Supplement: Supplementary file 1 — Supplementary Information. [file 41598_2021_99478_MOESM1_ESM.docx]

**Supplementary Information**

The Role of cardiovascular risk factors in maternal cardiovascular disease according to offspring birth characteristics in the HUNT study

**Authors:** Eirin B. Haug; Amanda R. Markovitz; Abigail Fraser; Håvard Dalen; Pål R. Romundstad; Bjørn O. Åsvold; Janet W. Rich-Edwards; Julie Horn

Supplementary Appendix S1. Cardiovascular risk factors

All serum analyses were performed in fresh non-fasting samples at the Central Laboratory, Levanger Hospital, Nord-Trøndelag Hospital Trust using a Hitachi 911 Autoanalyzer in HUNT2 and Architect cSystems ci8200 in HUNT3. Height and weight were measured with the person wearing light clothes and no shoes and were rounded to the nearest cm (height) and half kilo (weight). BMI was calculated as weight (in kg) divided by the squared value of height (in m). Blood pressure in HUNT1 was measured manually two times at 1-minute intervals using a sphygmomanometer after the person had come to rest, and we used the mean value of these two measurements in our analysis. In HUNT2 and HUNT3, blood pressure was measured three times at 1-minute intervals using an automatic oscillometric method (Dinamap, Critikon, Florida) after the person had come to rest, with cuff size adjusted to arm circumference. We used the mean of the second and third measurement, except for 2153 women in HUNT3 who lacked the third measurement due to sick leave amongst staff; for them, we used the second measurement only. Serum total and high-density lipoprotein (HDL) cholesterol and triglycerides were analyzed using enzymatic colorimetric methods (Boeheringer Mannheim, Germany) in HUNT2. In HUNT3 HDL cholesterol was measured with an accelerator selective detergent methodology, total cholesterol was analyzed by a cholesterol esterase methodology by equipment from Abbott, Clinical Chemistry, USA. Non-HDL cholesterol was calculated as the difference between total and HDL cholesterol. In HUNT1 capillary glucose was measured at the examination stations in participants above 40 years (Reflocheck-Glucose, Boehringer Mannheim, Germany), and for the analysis of mean glucose levels, we transformed capillary levels to equate serum values (in mmol/L) by multiplying with 1.11[5]. In HUNT2 and HUNT3 serum glucose was measured for all persons using an enzymatic hexokinase method.

Supplementary Appendix S2. Validation Information

From the electronic patient administrative system at the two primary hospitals in Nord-Trøndelag county, Levanger Hospital and Namsos Hospital (Nord-Trøndelag Hospital Trust), we obtained information on all study participants registered with at least one of the following cardiovascular diagnoses between September 1, 1987 (the first date of electronic recording) and April 24, 2015: ICD-9,402, 404, 410-414, 425, 427.5, 428, 430-438, 440-448; ICD-10, G45, I11, I13, I20-I25, I42, I46, I50, I60-I67, I71, I72, I74. For each identified patient, one of two experienced cardiologists (B.K., H.D.), who were unaware of the pregnancy history of the participants, examined the medical records to determine the first validated occurrence of each of the following cardiovascular events: myocardial infarction, heart failure, cardiac arrest, cerebral infarction, transient ischaemic attack (TIA), intracranial hemorrhage, aortic dissection or aneurysm, dissection or aneurysm of peripheral arteries, and embolism or thrombosis in peripheral arteries. Myocardial infarction diagnosed using ESC/ACCF/AHA/WHF criteria[6] and classified as STEMI or NSTEMI. Heart failure was diagnosed based on echocardiography findings combined with symptoms/signs according to ESC guidelines,[7] and was classified as systolic or diastolic. Ejection fraction was recorded or, if possible, estimated based on the echocardiography description in cases where no value was reported. If echocardiography had not been performed, the diagnosis was made using the clinically based Framingham criteria[8]. Right ventricular failure without left ventricular failure (e.g. cor pulmonale following pulmonary embolism or COPD) was not included. Cardiac arrest was diagnosed based on a documented or highly probable cardial cause of pulselessness and lack of breathing requiring cardiopulmonary resuscitation at hospital or before hospital admission. Cerebral infarction was diagnosed based on typical symptoms and signs combined with radiological evidence from computer tomography (CT) or magnetic resonance imaging (MRI) scans. In some cases, the diagnosis of cerebral infarction was based on typical symptoms and signs in absence of radiological evidence, but only if CT or MRI scan had been performed, and no alternative explanation for the clinical presentation was found. TIA was diagnosed based on typical symptoms/signs combined with absence of radiological signs of cerebral infarction on CT or MRI scan. Events probably caused by other diseases were excluded. TIA occurring after intracerebral infarction was not recorded. Possible cerebrovascular events accompanying intracerebral malignancy, infection or inflammation were not included. Transitory global amnesia and migraine with aura were not considered as valid cerebrovascular diagnoses. Intracranial hemorrhage was classified based on radiological evidence as subarachnoid, intracerebral, subdural or epidural, and hemorrhage secondary to trauma or intracerebral malignancy was not included. For a diagnosis of aortic aneurysm, an aortic diameter >30 mm in abdomen or >40 mm in thorax was required.

**References**

1. Holmen J, Midthjell K, Bjartveit K, Hjort PF, Lund-Larsen PG. The Nord-Trøndelag health Survey 1984-1986. Purpose, background and methods. Participation, non-participation and frequency distributions [Internet]. Verdal: Senter for samfunnsmedisinsk forskning, Statens Institutt for folkehelse(SIFF). Helsetjenesteforskning; 1990 p. 1–257. Available from: https://www.ntnu.no/c/document_library/get_file?uuid=4be1f10f-be02-484d-a033-6194748bd5f4&groupId=10304

2. Holmen J, Midthjell K, Krüger Ø, Langhammer A, Holmen TL, Bratberg G, et al. The Nord-Trøndelag Health Study 1995-97(HUNT2). Objectives contents, methods and participation. J Epid. 2003;19–32.

3. Krokstad S, Langhammer A, Hveem K, Holmen TL, Midthjell K, Stene TR, et al. Cohort Profile: the HUNT Study, Norway. Int J Epidemiol. 2013;968–77. https://doi.org/10.1093/ije/dys095.

4. Irgens LM. The medical birth Registry of Norway; a source for epidemiological and clinical research. Scand J Rheumatol. 1998;105–8. https://doi.org/10.1080/03009742.1998.11720780.

5. Sacks DB, Arnold M, Bakris GL, Bruns DE, Horvath AR, Kirkman MS, et al. Guidelines and Recommendations for Laboratory Analysis in the Diagnosis and Management of Diabetes Mellitus. Diabetes Care. 2011;e61–99. https://doi.org/10.2337/dc11-9998.

6. Thygesen K, Alpert JS, Jaffe AS, Simoons ML, Chaitman BR, White HD, et al. Third universal definition of myocardial infarction. Eur Heart J. 2012;2551–67. https://doi.org/10.1093/eurheartj/ehs184.

7. McMurray JJV, Adamopoulos S, Anker SD, Auricchio A, Böhm M, Dickstein K, et al. ESC Guidelines for the diagnosis and treatment of acute and chronic heart failure 2012: The Task Force for the Diagnosis and Treatment of Acute and Chronic Heart Failure 2012 of the European Society of Cardiology. Developed in collaboration with the Heart Failure Association (HFA) of the ESC. Eur Heart J. 2012;1787–847. https://doi.org/10.1093/eurheartj/ehs104.

8. McKee PA, Castelli WP, McNamara PM, Kannel WB. The natural history of congestive heart failure: the Framingham study. N Engl J Med. 1971;1441–6. https://doi.org/10.1056/NEJM197112232852601.

| **Supplementary Table S1. ICD codes for fatal cardiovascular events in the Cause of Death Registry** | | |
| --- | --- | --- |
|  | **ICD-9 codes**  **(1986-95)** | **ICD-10 codes**  **(from 1996)** |
| **All cardiovascular events** | 401-414 and 424-445 | G45, I10-I25, I34-I37, I42-I51, and I60-I77 |
| **Myocardial infarction** | 410 and 412 | I21, I22 and I25.2 |
| **Heart failure** | 425 and 428 | I42 and I50 |
| **Cerebrovascular disease** | 430, 431 and 433-435 | G45, I60, I61, I63, I64, and I69.0, .1, .3, and .4. |


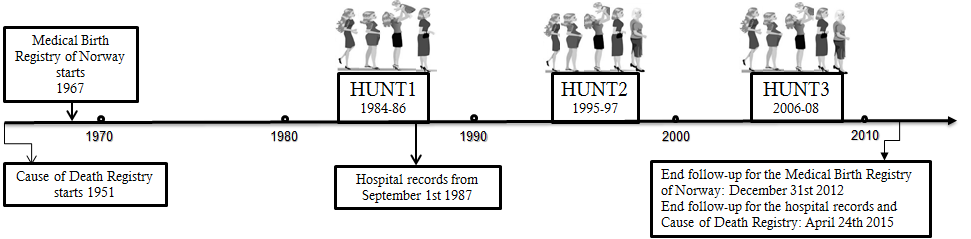


**Supplementary Figure S1.** Timeline of follow-up with data sources.


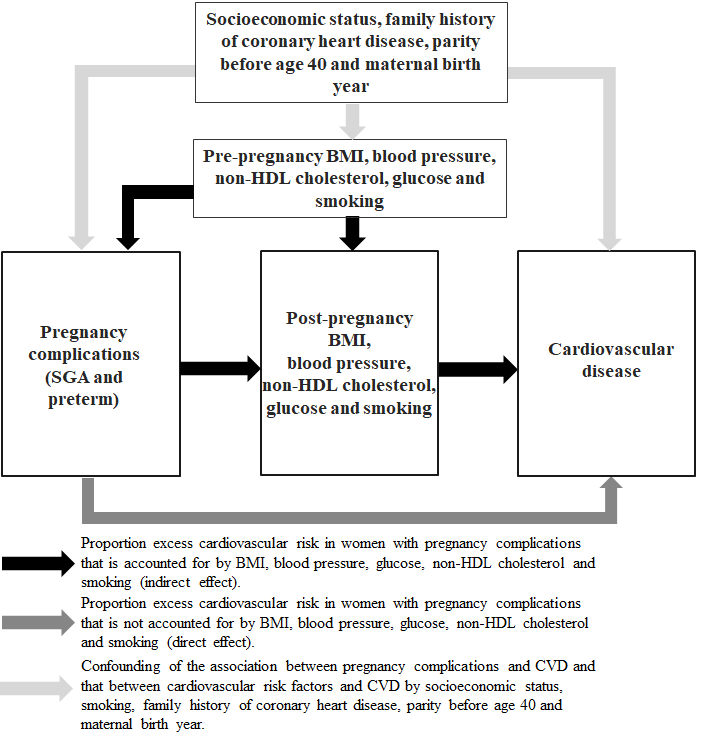


**Supplementary Figure S2**. Diagram of mediation analysis.

| **Supplementary Table S2. Descriptive characteristics of included pregnancies.** | | | | | | |
| --- | --- | --- | --- | --- | --- | --- |
|  | **Fetal Growth** | | |  | **Gestational length** | |
|  | SGA | AGA | LGA |  | Term | Preterm (<37 weeks) |
| Number of women | 4203 (18) | 15109 (65) | 3814 (16) |  | 21175 (91) | 2109 ( 9) |
| Number of pregnancies | 9672 (20) | 30159 (61) | 9411 (19) |  | 44462 (89) | 5262 (11) |
| Prepregancy or gestational diabetes (%) | |  |  |  |  |  |
| No | 9656 (100) | 30091 (100) | 9341 (99) |  | 44338 (100) | 5226 (99) |
| Yes | 16 (0.17) | 68 (0.23) | 70 (0.74) |  | 124 (0.28) | 36 (0.68) |
| till birth (%) |  |  |  |  |  |  |
| No | 9505 (98) | 30034 (100) | 9356 (99) |  | 44304 (100) | 5058 (96) |
| Yes | 167 (1.73) | 125 (0.41) | 55 (0.58) |  | 158 (0.36) | 204 (3.88) |
| Hypertensive disorders in pregnancy (%) | |  |  |  |  |  |
| No | 8972 (93) | 28969 (96) | 8954 (95) |  | 42505 (96) | 4833 (92) |
| Yes | 700 ( 7) | 1190 ( 4) | 457 ( 5) |  | 1957 ( 4) | 429 ( 8) |
| Preterm (<37 weeks) (%) |  |  |  |  | N/A | |
| No | 9021 (93) | 29011 (96) | 8941 (95) |  |  |  |
| Yes | 651 ( 7) | 1148 ( 4) | 470 ( 5) |  |  |  |
| Weight for gestational length (%) |  |  |  |  |  |  |
| Small for gestational age | N/A | | |  | 4474 (10) | 793 (15) |
| Appropriate for gestational age |  |  |  |  | 35563 (80) | 3869 (74) |
| Large for gestation age |  |  |  |  | 4425 (10) | 600 (11) |
| SGA = small for gestational age; AGA = appropriate size for gestational age; LGA = large for gestational age; N/A = not applicable | | | | | | |

| **Supplementary Table S3. Timing of ascertainment of the cardiovascular risk factors used in the analysis of the contribution of these risk factors to excess cardiovascular in women with history of SGA offspring.** | | |
| --- | --- | --- |
| **Cardiovascular risk factor** | Main analysis^1^ | Sensitivity analysis^2^ |
| **BMI** |  |  |
| Median age of measurement (IQR) | 50 (41 - 58) | 36 (30 - 41) |
| Measured before index pregnany (%) |  |  |
| Yes | 392 ( 2) | 1516 ( 8) |
| No | 18641 (98) | 17450 (92) |
| Median years between measurement and index pregnancy (IQR) | 26 (17 - 35) | 13 ( 7 - 17) |
| Median years between measurement and end of follow-up (IQR) | 8 ( 7 - 9) | 29 (19 - 31) |
| **SBP** |  |  |
| Median age of measurement (IQR) | 50 (41 - 58) | 36 (30 - 41) |
| Measured before index pregnany (%) |  |  |
| Yes | 393 ( 2) | 1518 ( 8) |
| No | 18628 (98) | 17448 (92) |
| Median years between measurement and index pregnancy (IQR) | 26 (17 - 35) | 13 ( 7 - 17) |
| Median years between measurement and end of follow-up (IQR) | 8 ( 7 - 9) | 29 (19 - 31) |
| **DBP** |  |  |
| Median age of measurement (IQR) | 50 (41 - 58) | 36 (30 - 41) |
| Measured before index pregnany (%) |  |  |
| Yes | 393 ( 2) | 1518 ( 8) |
| No | 18628 (98) | 17446 (92) |
| Median years between measurement and index pregnancy (IQR) | 26 (17 - 35) | 13 ( 7 - 17) |
| Median years between measurement and end of follow-up (IQR) | 8 ( 7 - 9) | 29 (19 - 31) |
| **Glucose** |  |  |
| Median age of measurement (IQR) | 51 (42 - 59) | 42 (36 - 47) |
| Measured before index pregnany (%) |  |  |
| Yes | 219 ( 1) | 569 ( 3) |
| No | 17464 (99) | 16779 (97) |
| Median years between measurement and index pregnancy (IQR) | 27 (18 - 36) | 17 (12 - 24) |
| Median years between measurement and end of follow-up (IQR) | 8 ( 7 - 9) | 19 (18 - 20) |
| **Non-HDL cholesterol** |  |  |
| Median age of measurement (IQR) | 51 (42 - 59) | 43 (36 - 50) |
| Measured before index pregnany (%) |  |  |
| Yes | 219 ( 1) | 569 ( 3) |
| No | 17169 (99) | 16413 (97) |
| Median years between measurement and index pregnancy (IQR) | 27 (18 - 36) | 21 (12 - 27) |
| Median years between measurement and end of follow-up (IQR) | 8 ( 7 - 9) | 19 (18 - 19) |
| **Smoking** |  |  |
| Median age of ascertainment (IQR) | 50 (41 - 58) | 35 (29 - 41) |
| Ascertained before index pregnancy (%) |  |  |
| Yes | 389 ( 2) | 1503 ( 9) |
| No | 18516 (98) | 15576 (91) |
| Median years between ascertainment and index pregnancy (IQR) | 26 (17 - 35) | 12 ( 6 - 16) |
| Median years between ascertainment and end of follow-up (IQR) | 8 ( 7 - 9) | 29 (19 - 31) |
| ^1^Analysis where cardiovascular risk factors were measured at the most recent HUNT exam. ^2^Analysis where cardiovascular risk factors were measured at the first HUNT exam. SGA = small for gestational age; BMI = body mass index; SBP = systolic blood pressure; DBP = diastolic blood pressure; HDL = high-density lipoprotein | | |
| **Supplementary Table S4. Timing of ascertainment of the cardiovascular risk factors used in the analysis of the contribution of these risk factors to excess cardiovascular in women with history of preterm birth.** | | |
| **Cardiovascular risk factor** | Main analysis^1^ | Sensitivity analysis^2^ |
| **BMI** |  |  |
| Median age of measurement (IQR) | 49 (41 - 58) | 36 (30 - 41) |
| Measured before index pregnany (%) |  |  |
| Yes | 480 ( 2) | 1838 ( 8) |
| No | 22465 (98) | 21024 (92) |
| Median years between measurement and index pregnancy (IQR) | 26 (17 - 35) | 12 ( 7 - 17) |
| Median years between measurement and end of follow-up (IQR) | 8 ( 7 - 9) | 29 (19 - 31) |
| **SBP** |  |  |
| Median age of measurement (IQR) | 49 (41 - 58) | 36 (30 - 41) |
| Measured before index pregnany (%) |  |  |
| Yes | 481 ( 2) | 1840 ( 8) |
| No | 22451 (98) | 21025 (92) |
| Median years between measurement and index pregnancy (IQR) | 26 (17 - 35) | 12 ( 7 - 17) |
| Median years between measurement and end of follow-up (IQR) | 8 ( 7 - 9) | 29 (19 - 31) |
| **DBP** |  |  |
| Median age of measurement (IQR) | 49 (41 - 58) | 36 (30 - 41) |
| Measured before index pregnany (%) |  |  |
| Yes | 481 ( 2) | 1840 ( 8) |
| No | 22452 (98) | 21021 (92) |
| Median years between measurement and index pregnancy (IQR) | 26 (17 - 35) | 12 ( 7 - 17) |
| Median years between measurement and end of follow-up (IQR) | 8 ( 7 - 9) | 29 (19 - 31) |
| **Glucose** |  |  |
| Median age of measurement (IQR) | 50 (42 - 59) | 42 (36 - 47) |
| Measured before index pregnany (%) |  |  |
| Yes | 276 ( 1) | 695 ( 3) |
| No | 21067 (99) | 20257 (97) |
| Median years between measurement and index pregnancy (IQR) | 27 (18 - 35) | 17 (12 - 23) |
| Median years between measurement and end of follow-up (IQR) | 8 ( 7 - 9) | 19 (18 - 20) |
| **Non-HDL cholesterol** |  |  |
| Median age of measurement (IQR) | 50 (42 - 59) | 43 (36 - 50) |
| Measured before index pregnany (%) |  |  |
| Yes | 276 ( 1) | 696 ( 3) |
| No | 20729 (99) | 19825 (97) |
| Median years between measurement and index pregnancy (IQR) | 27 (18 - 36) | 20 (12 - 27) |
| Median years between measurement and end of follow-up (IQR) | 8 ( 7 - 9) | 19 (18 - 19) |
| **Smoking** |  |  |
| Median age of ascertainment (IQR) | 49 (41 - 58) | 35 (29 - 41) |
| Ascertained before index pregnancy (%) |  |  |
| Yes | 477 ( 2) | 1812 ( 9) |
| No | 22312 (98) | 18816 (91) |
| Median years between ascertainment and index pregnancy (IQR) | 26 (17 - 35) | 12 ( 6 - 16) |
| Median years between ascertainment and end of follow-up (IQR) | 8 ( 7 - 9) | 29 (19 - 31) |
| ^1^Analysis where cardiovascular risk factors were measured at the most recent HUNT exam. ^2^Analysis where cardiovascular risk factors were measured at the first HUNT exam. BMI = body mass index; SBP = systolic blood pressure; DBP = diastolic blood pressure; HDL = high-density lipoprotein | | |

| **Supplementary Table S5. Hazard ratios for cardiovascular events in women with history of SGA or LGA offspring and whose first birth was recorded in the MBRN.** | | | | | | | | | |
| --- | --- | --- | --- | --- | --- | --- | --- | --- | --- |
|  |  |  |  |  | Model 1**^1^** | |  | Model 2^2^ | |
|  | No. of women | Person-years | No. of events |  | Hazard ratio (95% CI) | p-value |  | Hazard ratio (95% CI) | p-value |
| **Any CVD** |  |  |  |  |  |  |  |  |  |
| SGA | 3708 | 61556 | 207 |  | 1.23 (1.04 , 1.44) | 0.013 |  | 1.19 (1.01 , 1.40) | 0.038 |
| AGA | 12039 | 188585 | 500 |  | Ref. |  |  | Ref. |  |
| LGA | 3208 | 48944 | 105 |  | 0.83 (0.67 , 1.03) | 0.087 |  | 0.90 (0.73 , 1.12) | 0.338 |
| **Fatal CVD** |  |  |  |  |  |  |  |  |  |
| SGA | 3708 | 62853 | 20 |  | 1.57 (0.91 , 2.70) | 0.103 |  | 1.62 (0.93 , 2.81) | 0.087 |
| AGA | 12039 | 191576 | 38 |  | Ref. |  |  | Ref. |  |
| LGA | 3208 | 49538 | 6 |  | 0.64 (0.27 , 1.52) | 0.312 |  | 0.81 (0.34 , 1.95) | 0.646 |
| **Myocardial infarction** |  |  |  |  |  |  |  |  |  |
| SGA | 3708 | 62396 | 74 |  | 1.40 (1.06 , 1.84) | 0.018 |  | 1.30 (0.98 , 1.72) | 0.067 |
| AGA | 12039 | 190664 | 157 |  | Ref. |  |  | Ref. |  |
| LGA | 3208 | 49418 | 27 |  | 0.69 (0.46 , 1.03) | 0.070 |  | 0.77 (0.50 , 1.16) | 0.207 |
| **Heart failure** |  |  |  |  |  |  |  |  |  |
| SGA | 3708 | 62745 | 22 |  | 1.46 (0.88 , 2.44) | 0.145 |  | 1.45 (0.86 , 2.44) | 0.161 |
| AGA | 12039 | 191337 | 45 |  | Ref. |  |  | Ref. |  |
| LGA | 3208 | 49455 | 16 |  | 1.47 (0.83 , 2.60) | 0.187 |  | 1.67 (0.93 , 3.01) | 0.087 |
| **Cerebrovascular disease** |  |  |  |  |  |  |  |  |  |
| SGA | 3708 | 62164 | 109 |  | 1.18 (0.95 , 1.47) | 0.143 |  | 1.15 (0.92 , 1.44) | 0.224 |
| AGA | 12039 | 189831 | 272 |  | Ref. |  |  | Ref. |  |
| LGA | 3208 | 49161 | 56 |  | 0.82 (0.61 , 1.09) | 0.164 |  | 0.87 (0.65 , 1.17) | 0.352 |
| ^1^Adjusted for age. ^2^Adjusted for age, highest obtained educational level, parity before age 40, maternal birth year, maternal height and family history of coronary heart disease. SGA = small for gestational age; LGA = large for gestational age; MBRN = Medical Birth Registry of Norway; CI = confidence interval; CVD = cardiovascular disease, AGA = appropriate for gestational age | | | | | | | | | |

| **Supplementary Table S6. Hazard ratios for cardiovascular events according to SGA, AGA and LGA status of first birth.** | | | | | | | | | |
| --- | --- | --- | --- | --- | --- | --- | --- | --- | --- |
|  |  |  |  |  | Model 1**^1^** | |  | Model 2^2^ | |
|  | No. of women | Person-years | No. of events |  | Hazard ratio (95% CI) | p-value |  | Hazard ratio (95% CI) | p-value |
| **Any CVD** |  |  |  |  |  |  |  |  |  |
| SGA | 2585 | 41314 | 143 |  | 1.18 (0.98 , 1.41) | 0.077 |  | 1.12 (0.93 , 1.35) | 0.222 |
| AGA | 14654 | 223664 | 632 |  | Ref. |  |  | Ref. |  |
| LGA | 1063 | 15555 | 32 |  | 0.76 (0.53 , 1.08) | 0.128 |  | 0.78 (0.55 , 1.12) | 0.179 |
| **Fatal CVD** |  |  |  |  |  |  |  |  |  |
| SGA | 2585 | 42234 | 13 |  | 1.35 (0.73 , 2.49) | 0.335 |  | 1.28 (0.69 , 2.37) | 0.441 |
| AGA | 14654 | 227590 | 50 |  | Ref. |  |  | Ref. |  |
| LGA | 1063 | 15772 | 1 |  | 0.30 (0.04 , 2.19) | 0.236 |  | 0.32 (0.04 , 2.35) | 0.264 |
| **Myocardial infarction** |  |  |  |  |  |  |  |  |  |
| SGA | 14654 | 226454 | 193 |  | 1.23 (0.89 , 1.70) | 0.199 |  | 1.12 (0.81 , 1.55) | 0.506 |
| AGA | 2585 | 41911 | 46 |  | Ref. |  |  | Ref. |  |
| LGA | 1063 | 15720 | 10 |  | 0.78 (0.41 , 1.48) | 0.451 |  | 0.81 (0.43 , 1.54) | 0.524 |
| **Heart failure** |  |  |  |  |  |  |  |  |  |
| SGA | 2585 | 42146 | 14 |  | 1.12 (0.63 , 1.99) | 0.712 |  | 1.04 (0.58 , 1.87) | 0.897 |
| AGA | 14654 | 227254 | 64 |  | Ref. |  |  | Ref. |  |
| LGA | 1063 | 15749 | 4 |  | 0.97 (0.35 , 2.67) | 0.957 |  | 1.03 (0.37 , 2.85) | 0.951 |
| **Cerebrovascular disease** |  |  |  |  |  |  |  |  |  |
| SGA | 2585 | 41733 | 82 |  | 1.24 (0.98 , 1.58) | 0.078 |  | 1.19 (0.94 , 1.52) | 0.152 |
| AGA | 14654 | 225233 | 344 |  | Ref. |  |  | Ref. |  |
| LGA | 1063 | 15646 | 17 |  | 0.74 (0.45 , 1.20) | 0.218 |  | 0.76 (0.47 , 1.24) | 0.276 |
| ^1^Adjusted for age. ^2^Adjusted for age, highest obtained educational level, parity before age 40, maternal birth year, maternal height and family history of coronary heart disease. SGA = small for gestational age; LGA = large for gestational age; CI = confidence interval; CVD = cardiovascular disease,  AGA = appropriate for gestational age | | | | | | | | | |

| **Supplementary Table S7. Hazard ratios for cardiovascular events by history of SGA or LGA offspring, excluding women with history of stillbirth, gestational diabetes, chronic diabetes mellitus or hypertensive disorders.** | | | | | | | | | |
| --- | --- | --- | --- | --- | --- | --- | --- | --- | --- |
|  |  |  |  |  | Model 1**^1^** | |  | Model 2^2^ | |
|  | No. of women | Person-years | No. of events |  | Hazard ratio (95% CI) | p-value |  | Hazard ratio (95% CI) | p-value |
| **Any CVD** |  |  |  |  |  |  |  |  |  |
| SGA | 3451 | 59894 | 266 |  | 1.32 (1.15 , 1.52) | <0.001 |  | 1.33 (1.16 , 1.53) | <0.001 |
| AGA | 13703 | 237170 | 965 |  | Ref. |  |  | Ref. |  |
| LGA | 3269 | 54820 | 170 |  | 0.82 (0.69 , 0.96) | 0.015 |  | 0.85 (0.72 , 1.00) | 0.055 |
| **Fatal CVD** |  |  |  |  |  |  |  |  |  |
| SGA | 3451 | 61693 | 40 |  | 1.80 (1.25 , 2.58) | 0.001 |  | 1.97 (1.36 , 2.85) | <0.001 |
| AGA | 13703 | 243395 | 121 |  | Ref. |  |  | Ref. |  |
| LGA | 3269 | 55774 | 18 |  | 0.72 (0.44 , 1.18) | 0.192 |  | 0.77 (0.47 , 1.27) | 0.304 |
| **Myocardial infarction** |  |  |  |  |  |  |  |  |  |
| SGA | 3451 | 60971 | 92 |  | 1.39 (1.10 , 1.76) | 0.006 |  | 1.39 (1.09 , 1.76) | 0.007 |
| AGA | 13703 | 241243 | 315 |  | Ref. |  |  | Ref. |  |
| LGA | 3269 | 55498 | 47 |  | 0.70 (0.51 , 0.95) | 0.021 |  | 0.76 (0.56 , 1.04) | 0.089 |
| **Heart failure** |  |  |  |  |  |  |  |  |  |
| SGA | 3451 | 61544 | 33 |  | 1.41 (0.96 , 2.08) | 0.078 |  | 1.49 (1.01 , 2.21) | 0.046 |
| AGA | 13703 | 242784 | 129 |  | Ref. |  |  | Ref. |  |
| LGA | 3269 | 55637 | 29 |  | 1.09 (0.73 , 1.63) | 0.671 |  | 1.07 (0.71 , 1.61) | 0.760 |
| **Cerebrovascular disease** |  |  |  |  |  |  |  |  |  |
| SGA | 3451 | 60794 | 140 |  | 1.31 (1.09 , 1.58) | 0.005 |  | 1.30 (1.07 , 1.57) | 0.008 |
| AGA | 13703 | 240050 | 507 |  | Ref. |  |  | Ref. |  |
| LGA | 3269 | 55255 | 86 |  | 0.79 (0.63 , 0.99) | 0.043 |  | 0.83 (0.66 , 1.04) | 0.111 |
| ^1^Adjusted for age. ^2^Adjusted for age, highest obtained educational level, parity before age 40, maternal birth year, maternal height and family history of coronary heart disease. SGA = small for gestational age; LGA = large for gestational age; CI = confidence interval; CVD = cardiovascular disease, AGA=appropriate for gestational age | | | | | | | | | |

| **Supplementary Table S8. Hazard ratios for validated cardiovascular events by history of SGA or LGA offspring.** | | | | | | | | | |
| --- | --- | --- | --- | --- | --- | --- | --- | --- | --- |
|  |  |  |  |  | Model 1**^1^** | |  | Model 2^2^ | |
|  | No. of women | Person-years | No. of events |  | Hazard ratio (95% CI) | p-value |  | Hazard ratio (95% CI) | p-value |
| **Myocardial infarction** |  |  |  |  |  |  |  |  |  |
| SGA | 3745 | 66051 | 105 |  | 1.36 (1.09 , 1.69) | 0.006 |  | 1.37 (1.08 , 1.73) | 0.009 |
| AGA | 14712 | 257843 | 362 |  | Ref. |  |  | Ref. |  |
| LGA | 3463 | 58358 | 56 |  | 0.74 (0.56 , 0.98) | 0.034 |  | 0.77 (0.57 , 1.05) | 0.101 |
| **Heart failure** |  |  |  |  |  |  |  |  |  |
| SGA | 3745 | 66682 | 39 |  | 1.43 (1.00 , 2.05) | 0.048 |  | 1.43 (0.97 , 2.12) | 0.072 |
| AGA | 14712 | 259619 | 148 |  | Ref. |  |  | Ref. |  |
| LGA | 3463 | 58532 | 30 |  | 1.00 (0.68 , 1.49) | 0.980 |  | 1.05 (0.70 , 1.59) | 0.799 |
| **Cerebrovascular disease** |  |  |  |  |  |  |  |  |  |
| SGA | 3745 | 65845 | 156 |  | 1.30 (1.09 , 1.56) | 0.004 |  | 1.29 (1.07 , 1.56) | 0.008 |
| AGA | 14712 | 256675 | 560 |  | Ref. |  |  | Ref. |  |
| LGA | 3463 | 58136 | 93 |  | 0.79 (0.63 , 0.98) | 0.034 |  | 0.82 (0.65 , 1.03) | 0.092 |
| ^1^Adjusted for age. ^2^Adjusted for age, highest obtained educational level, parity before age 40, maternal birth year, maternal height and family history of coronary heart disease. SGA = small for gestational age; LGA = large for gestational age; CI = confidence interval; CVD = cardiovascular disease, AGA = appropriate for gestational age | | | | | | | | | |

| **Supplementary Table S9. Hazard ratios for cardiovascular events in women with history of severe SGA offspring** | | | | | | | | | |
| --- | --- | --- | --- | --- | --- | --- | --- | --- | --- |
|  |  |  |  |  | Model 1**^1^** | |  | Model 2^2^ | |
|  | No. of women | Person-years | No. of events |  | Hazard ratio  (95% CI) | p-value |  | Hazard ratio  (95% CI) | p-value |
| **Any CVD** |  |  |  |  |  |  |  |  |  |
| AGA | 15109 | 260172 | 1068 |  | Ref. |  |  | Ref. |  |
| SGA | 2276 | 39470 | 193 |  | 1.39 (1.20 , 1.63) | <0.001 | | 1.40 (1.20 , 1.64) | <0.001 |
| **Fatal CVD** |  |  |  |  |  |  |  |  |  |
| AGA | 15109 | 267087 | 135 |  | Ref. |  |  | Ref. |  |
| SGA | 2276 | 40860 | 30 |  | 1.87 (1.25 , 2.78) | 0.002 |  | 2.05 (1.37 , 3.08) | 0.001 |
| **Myocardial infarction** |  |  |  |  |  |  |  |  |  |
| AGA | 15109 | 264698 | 348 |  | Ref. |  |  | Ref. |  |
| SGA | 2276 | 40330 | 67 |  | 1.45 (1.12 , 1.89) | 0.005 |  | 1.42 (1.08 , 1.85) | 0.011 |
| **Heart failure** |  |  |  |  |  |  |  |  |  |
| AGA | 15109 | 266402 | 144 |  | Ref. |  |  | Ref. |  |
| SGA | 2276 | 40728 | 28 |  | 1.67 (1.11 , 2.51) | 0.013 |  | 1.81 (1.19 , 2.74) | 0.005 |
| **Cerebrovascular disease** |  |  |  |  |  |  |  |  |  |
| AGA | 15109 | 263363 | 558 |  | Ref. |  |  | Ref. |  |
| SGA | 2276 | 40151 | 100 |  | 1.35 (1.09 , 1.68) | 0.005 |  | 1.36 (1.10 , 1.70) | 0.005 |
| ^1^Adjusted for age. ^2^Adjusted for age, highest obtained educational level, parity before age 40, maternal birth year, maternal height and family history of coronary heart disease. SGA = small for gestational age defined as birth weight for gestational age and sex below the 5th percentile; CI = confidence interval; CVD = cardiovascular disease; AGA = appropriate for gestational age | | | | | | | | | |

| **Supplementary Table 10. Hazard ratios for cardiovascular events in women with history of SGA or LGA offspring and without history of preterm birth.** | | | | | | | | | |
| --- | --- | --- | --- | --- | --- | --- | --- | --- | --- |
|  |  |  |  |  | Model 1**^1^** | |  | Model 2**^2^** | |
|  | No. of women | Person-years | No. of events |  | Hazard ratio | p-value |  | Hazard ratio | p-value |
| **Any CVD** |  |  |  |  |  |  |  |  |  |
| SGA | 3630 | 63392 | 286 |  | 1.29 (1.13 , 1.47) | <0.001 | | 1.30 (1.14 , 1.49) | <0.001 |
| AGA | 14066 | 242570 | 996 |  | Ref. |  |  | Ref. |  |
| LGA | 3380 | 55899 | 177 |  | 0.82 (0.70 , 0.97) | 0.018 |  | 0.85 (0.72 , 1.00) | 0.055 |
| **Fatal CVD** |  |  |  |  |  |  |  |  |  |
| SGA | 3630 | 65387 | 44 |  | 1.78 (1.26 , 2.52) | 0.001 |  | 2.02 (1.42 , 2.88) | <0.001 |
| AGA | 14066 | 249072 | 124 |  | Ref. |  |  | Ref. |  |
| LGA | 3380 | 56935 | 17 |  | 0.66 (0.40 , 1.10) | 0.108 |  | 0.71 (0.43 , 1.19) | 0.197 |
| **Myocardial infarction** |  |  |  |  |  |  |  |  |  |
| SGA | 3630 | 64561 | 102 |  | 1.40 (1.12 , 1.75) | 0.003 |  | 1.41 (1.12 , 1.77) | 0.003 |
| AGA | 14066 | 246818 | 325 |  | Ref. |  |  | Ref. |  |
| LGA | 3380 | 56603 | 57 |  | 0.82 (0.62 , 1.09) | 0.165 |  | 0.90 (0.67 , 1.19) | 0.456 |
| **Heart failure** |  |  |  |  |  |  |  |  |  |
| SGA | 3630 | 65193 | 39 |  | 1.46 (1.02 , 2.09) | 0.038 |  | 1.50 (1.04 , 2.16) | 0.029 |
| AGA | 14066 | 248432 | 136 |  | Ref. |  |  | Ref. |  |
| LGA | 3380 | 56768 | 35 |  | 1.25 (0.86 , 1.82) | 0.235 |  | 1.25 (0.86 , 1.83) | 0.247 |
| **Cerebrovascular disease** |  |  |  |  |  |  |  |  |  |
| SGA | 3630 | 64416 | 149 |  | 1.26 (1.05 , 1.52) | 0.012 |  | 1.25 (1.04 , 1.50) | 0.019 |
| AGA | 14066 | 245577 | 524 |  | Ref. |  |  | Ref. |  |
| LGA | 3380 | 56388 | 88 |  | 0.78 (0.62 , 0.98) | 0.033 |  | 0.81 (0.64 , 1.02) | 0.068 |
| ^1^Adjusted for age. ^2^Adjusted for age, highest obtained educational level, parity before age 40, maternal birth year, maternal height and family history of coronary heart disease. SGA = small for gestational age; CI = confidence interval; CVD = cardiovascular disease; AGA = appropriate for gestational age; LGA = large for gestational age | | | | | | | | | |

| **Supplementary Table 11. Hazard ratios for cardiovascular events in women with history of term or preterm birth and SGA offspring.** | | | | | | | | | |
| --- | --- | --- | --- | --- | --- | --- | --- | --- | --- |
|  |  |  |  |  | Model 1**^1^** | |  | Model 2**^2^** | |
|  | No. of women | Person-years | No. of events |  | Hazard ratio | p-value |  | Hazard ratio | p-value |
| **Any CVD** |  |  |  |  |  |  |  |  |  |
| AGA + term | 14140 | 243492 | 1001 |  | Ref. |  |  | Ref. |  |
| SGA + term | 3752 | 65160 | 294 |  | 1.28 (1.13 , 1.46) | <0.001 |  | 1.30 (1.14 , 1.48) | <0.001 |
| SGA + preterm | 451 | 7193 | 35 |  | 1.53 (1.09 , 2.14) | 0.014 |  | 1.53 (1.09 , 2.16) | 0.015 |
| **Fatal CVD** |  |  |  |  |  |  |  |  |  |
| AGA + term | 14140 | 250035 | 125 |  | Ref. |  |  | Ref. |  |
| SGA + term | 3752 | 67237 | 44 |  | 1.72 (1.21 , 2.43) | 0.002 |  | 1.92 (1.35 , 2.74) | <0.001 |
| SGA + preterm | 451 | 7402 | 4 |  | 1.66 (0.61 , 4.49) | 0.322 |  | 2.06 (0.75 , 5.69) | 0.162 |
| **Myocardial infarction** |  |  |  |  |  |  |  |  |  |
| AGA + term | 14140 | 247771 | 326 |  | Ref. |  |  | Ref. |  |
| SGA + term | 3752 | 66370 | 106 |  | 1.40 (1.13 , 1.75) | 0.003 |  | 1.41 (1.12 , 1.77) | 0.003 |
| SGA + preterm | 451 | 7344 | 11 |  | 1.45 (0.79 , 2.65) | 0.226 |  | 1.44 (0.78 , 2.65) | 0.243 |
| **Heart failure** |  |  |  |  |  |  |  |  |  |
| AGA + term | 14140 | 249372 | 138 |  | Ref. |  |  | Ref. |  |
| SGA + term | 3752 | 67043 | 39 |  | 1.41 (0.98 , 2.02) | 0.061 |  | 1.49 (1.03 , 2.14) | 0.034 |
| SGA + preterm | 451 | 7391 | 3 |  | 1.19 (0.38 , 3.73) | 0.770 |  | 1.28 (0.40 , 4.09) | 0.675 |
| **Cerebrovascular disease** |  |  |  |  |  |  |  |  |  |
| AGA + term | 14140 | 246534 | 525 |  | Ref. |  |  | Ref. |  |
| SGA + term | 3752 | 66219 | 155 |  | 1.28 (1.07 , 1.53) | 0.008 |  | 1.28 (1.06 , 1.54) | 0.009 |
| SGA + preterm | 451 | 7288 | 18 |  | 1.50 (0.94 , 2.40) | 0.092 |  | 1.51 (0.94 , 2.44) | 0.090 |
| ^1^Adjusted for age. ^2^Adjusted for age, highest obtained educational level, parity before age 40, maternal birth year, maternal height and family history of coronary heart disease. SGA + term = history of small for gestational age and no preterm births; SGA + preterm = history of SGA offspring and preterm birth (<37 weeks); CI = confidence interval; CVD = cardiovascular disease; AGA + term = history of appropriate for gestational age offspring and no preterm births | | | | | | | | | |

| **Supplementary Table 12. Hazard ratios for cardiovascular events in women with history of term or preterm birth and LGA offspring.** | | | | | | | | | |
| --- | --- | --- | --- | --- | --- | --- | --- | --- | --- |
|  |  |  |  |  | Model 1**^1^** | |  | Model 2**^2^** | |
|  | No. of women | Person-years | No. of events |  | Hazard ratio | p-value |  | Hazard ratio | p-value |
| **Any CVD** |  |  |  |  |  |  |  |  |  |
| AGA + term | 14140 | 243492 | 1001 |  | Ref. |  |  | Ref. |  |
| LGA + term | 3405 | 56223 | 180 |  | 0.83 (0.71 , 0.98) | 0.023 |  | 0.85 (0.72 , 0.99) | 0.043 |
| LGA + preterm | 409 | 7011 | 27 |  | 1.14 (0.78 , 1.68) | 0.494 |  | 1.18 (0.80 , 1.73) | 0.403 |
| **Fatal CVD** |  |  |  |  |  |  |  |  |  |
| AGA + term | 14140 | 250035 | 125 |  | Ref. |  |  | Ref. |  |
| LGA + term | 3405 | 57287 | 17 |  | 0.65 (0.39 , 1.08) | 0.098 |  | 0.72 (0.43 , 1.20) | 0.207 |
| LGA + preterm | 409 | 7132 | 5 |  | 2.05 (0.84 , 5.03) | 0.116 |  | 2.71 (1.09 , 6.75) | 0.032 |
| **Myocardial infarction** |  |  |  |  |  |  |  |  |  |
| AGA + term | 14140 | 247771 | 326 |  | Ref. |  |  | Ref. |  |
| LGA + term | 3405 | 56954 | 58 |  | 0.83 (0.63 , 1.10) | 0.192 |  | 0.89 (0.67 , 1.19) | 0.439 |
| LGA + preterm | 409 | 7105 | 7 |  | 0.91 (0.43 , 1.93) | 0.813 |  | 1.04 (0.49 , 2.20) | 0.926 |
| **Heart failure** |  |  |  |  |  |  |  |  |  |
| AGA + term | 14140 | 249372 | 138 |  | Ref. |  |  | Ref. |  |
| LGA + term | 3405 | 57121 | 35 |  | 1.23 (0.85 , 1.78) | 0.280 |  | 1.23 (0.84 , 1.80) | 0.289 |
| LGA + preterm | 409 | 7119 | 3 |  | 1.08 (0.34 , 3.39) | 0.897 |  | 1.14 (0.36 , 3.62) | 0.825 |
| **Cerebrovascular disease** |  |  |  |  |  |  |  |  |  |
| AGA + term | 14140 | 246534 | 525 |  | Ref. |  |  | Ref. |  |
| LGA + term | 3405 | 56730 | 89 |  | 0.79 (0.63 , 0.98) | 0.036 |  | 0.79 (0.63 , 1.00) | 0.049 |
| LGA + preterm | 409 | 7043 | 14 |  | 1.15 (0.67 , 1.95) | 0.615 |  | 1.14 (0.66 , 1.94) | 0.639 |
| ^1^Adjusted for age. ^2^Adjusted for age, highest obtained educational level, parity before age 40, maternal birth year, maternal height and family history of coronary heart disease. LGA + term = history of large for gestational age and no preterm births; LGA + preterm = history of LGA offspring and preterm birth (<37 weeks); AGA + term = history of appropriate for gestational age offspring and no preterm births; CI = confidence interval | | | | | | | | | |

| **Supplementary Table S13. Decomposition of the association between history of SGA offspring and cardiovascular disease into direct and indirect effects among women who had their cardiovascular risk factors measured or collected after age 40.** | | | | | | | | | | | | |
| --- | --- | --- | --- | --- | --- | --- | --- | --- | --- | --- | --- | --- |
|  |  |  | Total effect^1^ | | | Direct effect^2^ | |  | Indirect effect^3^ | |  | Proportion explained^4^ |
| Cardiovascular risk factors | No. of women |  | HR (95% CI) | p-value |  | HR (95% CI) | p-value |  | HR (95% CI) | p-value |  | Percentage |
| BMI^5^ | 14660 |  | 1.24 (1.08 , 1.43) | 0.002 |  | 1.27 (1.09 , 1.48) | 0.003 |  | 0.98 (0.92 , 1.05) | 0.570 |  | -9 |
| Systolic blood pressure^5^ | 14653 |  | 1.24 (1.07 , 1.44) | 0.004 |  | 1.22 (1.04 , 1.43) | 0.014 |  | 1.02 (0.96 , 1.08) | 0.560 |  | 8 |
| Diastolic blood pressure^5^ | 14654 |  | 1.24 (1.07 , 1.43) | 0.003 |  | 1.23 (1.06 , 1.44) | 0.008 |  | 1.01 (0.95 , 1.06) | 0.855 |  | 2 |
| Serum glucose^5^ | 14330 |  | 1.25 (1.08 , 1.45) | 0.003 |  | 1.26 (1.07 , 1.48) | 0.004 |  | 1.00 (0.94 , 1.05) | 0.883 |  | -2 |
| Serum non-HDL cholesterol^5^ | 14036 |  | 1.23 (1.06 , 1.43) | 0.006 |  | 1.24 (1.05 , 1.46) | 0.010 |  | 1.00 (0.94 , 1.06) | 0.924 |  | -1 |
| Smoking^6^ | 14564 |  | 1.32 (1.15 , 1.52) | <0.001 |  | 1.19 (1.02 , 1.39) | 0.024 |  | 1.11 (1.04 , 1.18) | 0.001 |  | 37 |
| ^1^Association between history of SGA offspring and CVD compared to women with history of appropriate size for age offspring. ^2^Part of the association between history of SGA offspring and CVD that is not explained by the cardiovascular risk factor. ^3^Part of the association between history of SGA offspring and CVD that is explained by the cardiovascular risk factor. ^4^Proportion excess cardiovascular risk in women with history of SGA offspring that is explained by cardiovascular risk factor.  ^5^Estimates are adjusted for age (used as time scale in the Cox proportional hazards model), age at measurement of the cardiovascular risk factor, highest obtained educational level, age at first birth, parity, first birth in the Medical Birth Registry of Norway, maternal height and birth year. ^6^Estimates are adjusted for age (used as time scale in the Cox proportional hazards model), highest obtained educational level, age at first birth, parity, first birth in the Medical Birth Registry of Norway, maternal height and birth year. SGA = small for gestational age; CVD = cardiovascular disease; CI = confidence interval, BMI = body mass index; HDL = high-density lipoprotein | | | | | | | | | | | | |
|  | | | | | | | | | | | | |

| **Supplementary Table S14. Decomposition of the association between history of SGA offspring and cardiovascular disease into direct and indirect effects in women without history of hypertensive disorders of pregnancy, gestational diabetes, chronic diabetes mellitus and stillbirth.** | | | | | | | | | | | | |
| --- | --- | --- | --- | --- | --- | --- | --- | --- | --- | --- | --- | --- |
|  | No. of women |  | Total effect^1^ | |  | Direct effect^2^ | |  | Indirect effect^3^ | |  | Proportion explained^4^ |
| Cardiovascular risk factor |  |  | HR (95% CI) | p-value |  | HR (95% CI) | p-value |  | HR (95% CI) | p-value |  | Percentage |
| BMI^5^ | 16921 |  | 1.24 (1.07 , 1.44) | 0.005 |  | 1.25 (1.06 , 1.47) | 0.007 |  | 0.99 (0.93 , 1.06) | 0.778 |  | -4 |
| Systolic blood pressure^5^ | 16910 |  | 1.24 (1.08 , 1.44) | 0.003 |  | 1.23 (1.05 , 1.43) | 0.009 |  | 1.01 (0.96 , 1.07) | 0.616 |  | 7 |
| Diastolic blood pressure^5^ | 16910 |  | 1.24 (1.08 , 1.44) | 0.003 |  | 1.23 (1.05 , 1.44) | 0.009 |  | 1.01 (0.95 , 1.07) | 0.729 |  | 5 |
| Serum glucose^5^ | 15743 |  | 1.26 (1.08 , 1.47) | 0.004 |  | 1.28 (1.08 , 1.51) | 0.005 |  | 0.99 (0.93 , 1.05) | 0.658 |  | -6 |
| Serum non-HDL cholesterol^5^ | 15481 |  | 1.25 (1.07 , 1.45) | 0.005 |  | 1.26 (1.07 , 1.48) | 0.005 |  | 0.99 (0.93 , 1.05) | 0.708 |  | -6 |
| Smoking^6^ | 16937 |  | 1.31 (1.14 , 1.52) | <0.001 |  | 1.14 (0.97 , 1.34) | 0.120 |  | 1.15 (1.07 , 1.24) | <0.001 |  | 53 |
| ^1^Association between history of SGA offspring and CVD compared to women with history of appropriate size for age offspring. ^2^Part of the association between history of SGA offspring and CVD that is not explained by the cardiovascular risk factor. ^3^Part of the association between history of SGA offspring and CVD that is explained by the cardiovascular risk factor. ^4^Proportion excess cardiovascular risk in women with history of SGA offspring that is explained by cardiovascular risk factor. ^5^Estimates are adjusted for age (used as time scale in the Cox proportional hazards model), age at measurement of the cardiovascular risk factor, highest obtained educational level, age at first birth, parity, first birth in the Medical Birth Registry of Norway, maternal height and birth year. ^6^Estimates are adjusted for age (used as time scale in the Cox proportional hazards model), highest obtained educational level, age at first birth, parity, first birth in the Medical Birth Registry of Norway, maternal height and birth year. CVD = cardiovascular disease; CI = confidence interval, BMI = body mass index; HDL = high-density lipoprotein | | | | | | | | | | | | |

| **Supplementary Table S15. Decomposition of the association between history of severe SGA^1^ offspring and cardiovascular disease into direct and indirect effects.** | | | | | | | | | | | | |
| --- | --- | --- | --- | --- | --- | --- | --- | --- | --- | --- | --- | --- |
|  | No. of women |  | Total effect^2^ | |  | Direct effect^3^ | |  | Indirect effect^4^ | |  | Proportion explained^5^ |
| Cardiovascular risk factor |  |  | HR (95% CI) | p-value |  | HR (95% CI) | p-value |  | HR (95% CI) | p-value |  | Percentage |
| BMI^6^ | 17136 |  | 1.31 (1.12 , 1.53) | 0.001 |  | 1.30 (1.10 , 1.55) | 0.003 |  | 1.00 (0.93 , 1.09) | 0.920 |  | 2 |
| Systolic blood pressure^6^ | 17120 |  | 1.31 (1.12 , 1.54) | 0.001 |  | 1.25 (1.04 , 1.50) | 0.017 |  | 1.05 (0.97 , 1.14) | 0.213 |  | 19 |
| Diastolic blood pressure^6^ | 17120 |  | 1.31 (1.12 , 1.54) | 0.001 |  | 1.26 (1.06 , 1.51) | 0.010 |  | 1.04 (0.96 , 1.13) | 0.347 |  | 14 |
| Serum glucose^6^ | 15948 |  | 1.31 (1.10 , 1.56) | 0.002 |  | 1.31 (1.08 , 1.58) | 0.006 |  | 1.00 (0.92 , 1.09) | 0.968 |  | 1 |
| Serum non-HDL cholesterol^6^ | 15677 |  | 1.33 (1.11 , 1.58) | 0.002 |  | 1.33 (1.09 , 1.62) | 0.005 |  | 1.00 (0.92 , 1.08) | 0.917 |  | -2 |
| Smoking^7^ | 17016 |  | 1.35 (1.15 , 1.58) | <0.001 |  | 1.17 (0.97 , 1.40) | 0.095 |  | 1.16 (1.06 , 1.26) | 0.001 |  | 48 |
| ^1^Severe SGA = small for gestational age defined as birth weight for gestational age and sex below the 5th percentile. ^2^Association between history of SGA offspring and CVD compared to women with history of appropriate size for age offspring. ^3^Part of the association between history of SGA offspring and CVD that is not explained by the cardiovascular risk factor. ^4^Part of the association between history of SGA offspring and CVD that is explained by the cardiovascular risk factor. ^5^Proportion excess cardiovascular risk in women with history of SGA offspring that is explained by cardiovascular risk factor. ^6^Estimates are adjusted for age (used as time scale in the Cox proportional hazards model), age at measurement of the cardiovascular risk factor, highest obtained educational level, age at first birth, parity, first birth in the Medical Birth Registry of Norway, maternal height and birth year. ^7^Estimates are adjusted for age (used as time scale in the Cox proportional hazards model), highest obtained educational level, age at first birth, parity, first birth in the Medical Birth Registry of Norway, maternal height and birth year.  CVD = cardiovascular disease; CI = confidence interval, BMI = body mass index; HDL = high-density lipoprotein | | | | | | | | | | | | |

| **Supplementary Table S16. Decomposition of the association between history of SGA offspring and cardiovascular disease into direct and indirect effect where cardiovascular risk factors were measured at women’s first HUNT exam.** | | | | | | | | | | | | |
| --- | --- | --- | --- | --- | --- | --- | --- | --- | --- | --- | --- | --- |
|  | No. of women |  | Total effect^1^ | |  | Direct effect of HDP^2^ | |  | Indirect effect^3^ | |  | Proportion explained^4^ |
| Cardiovascular risk factor |  |  | HR (95% CI) | p-value |  | HR (95% CI) | p-value |  | HR (95% CI) | p-value |  | Percentage |
| BMI^5^ | 18966 |  | 1.32 (1.16 , 1.50) | <0.001 |  | 1.38 (1.20 , 1.58) | <0.001 |  | 0.96 (0.91 , 1.01) | 0.138 |  | -15 |
| Systolic blood pressure^5^ | 18966 |  | 1.32 (1.16 , 1.51) | <0.001 |  | 1.30 (1.13 , 1.50) | <0.001 |  | 1.01 (0.96 , 1.07) | 0.621 |  | 5 |
| Diastolic blood pressure^5^ | 18964 |  | 1.32 (1.16 , 1.50) | <0.001 |  | 1.31 (1.14 , 1.51) | <0.001 |  | 1.01 (0.96 , 1.06) | 0.776 |  | 3 |
| Serum glucose^5^ | 17348 |  | 1.33 (1.16 , 1.52) | <0.001 |  | 1.34 (1.16 , 1.54) | <0.001 |  | 0.99 (0.94 , 1.04) | 0.750 |  | -3 |
| Serum non-HDL cholesterol^5^ | 16982 |  | 1.30 (1.12 , 1.50) | <0.001 |  | 1.29 (1.10 , 1.51) | 0.002 |  | 1.01 (0.95 , 1.07) | 0.764 |  | 3 |
| Smoking^6^ | 17079 |  | 1.31 (1.14 , 1.50) | <0.001 |  | 1.14 (0.98 , 1.33) | 0.078 |  | 1.15 (1.08 , 1.22) | 0.000 |  | 50 |
| ^1^Association between history of SGA offspring and CVD compared to women with history of appropriate size for age offspring. ^2^Part of the association between history of SGA offspring and CVD that is not explained by the cardiovascular risk factor. ^3^Part of the association between history of SGA offspring and CVD that is explained by the cardiovascular risk factor. ^4^Proportion excess cardiovascular risk in women with history of SGA offspring that is explained by cardiovascular risk factor. ^5^Estimates are adjusted for age (used as time scale in the Cox proportional hazards model), age at measurement of the cardiovascular risk factor, highest obtained educational level, age at first birth, parity, first birth in the Medical Birth Registry of Norway, maternal height and birth year. ^6^Estimates are adjusted for age (used as time scale in the Cox proportional hazards model), age at first birth, parity, first birth in the Medical Birth Registry of Norway, maternal height and birth year. SGA = small for gestational age; CVD = cardiovascular disease; CI = confidence interval, BMI=body mass index; HDL = high-density lipoprotein | | | | | | | | | | | | |

| **Supplementary Table S17. Hazard ratios for cardiovascular events in women with history of very and moderate preterm birth** | | | | | | | | | |
| --- | --- | --- | --- | --- | --- | --- | --- | --- | --- |
|  |  |  |  |  | Model 1^1^ | |  | Model 2^2^ | |
|  | No. of women | Person-years | No. of events |  | Hazard ratio (95% CI) | p-value |  | Hazard ratio (95% CI) | p-value |
| **Any CVD** |  |  |  |  |  |  |  |  |  |
| Term | 21175 | 363529 | 1464 |  | Ref. |  |  | Ref. |  |
| 32-36 weeks | 1716 | 28188 | 124 |  | 1.25 (1.04 , 1.51) | 0.016 |  | 1.27 (1.06 , 1.53) | 0.011 |
| <32 weeks | 393 | 6719 | 26 |  | 1.11 (0.75 , 1.63) | 0.605 |  | 1.17 (0.79 , 1.73) | 0.420 |
| **Fatal CVD** |  |  |  |  |  |  |  |  |  |
| Term | 21175 | 373086 | 185 |  | Ref. |  |  | Ref. |  |
| 32-36 weeks | 1716 | 28879 | 18 |  | 1.60 (0.98 , 2.60) | 0.058 |  | 1.73 (1.06 , 2.82) | 0.028 |
| <32 weeks | 393 | 6913 | 3 |  | 1.11 (0.35 , 3.47) | 0.862 |  | 1.27 (0.40 , 4.01) | 0.681 |
| **Myocardial infarction** |  |  |  |  |  |  |  |  |  |
| Term | 21175 | 369670 | 486 |  | Ref. |  |  | Ref. |  |
| 32-36 weeks | 1716 | 28645 | 40 |  | 1.22 (0.88 , 1.68) | 0.227 |  | 1.26 (0.91 , 1.74) | 0.167 |
| <32 weeks | 393 | 6883 | 7 |  | 0.87 (0.41 , 1.82) | 0.704 |  | 0.95 (0.45 , 2.00) | 0.885 |
| **Heart failure** |  |  |  |  |  |  |  |  |  |
| Term | 21175 | 372086 | 210 |  | Ref. |  |  | Ref. |  |
| 32-36 weeks | 1716 | 28820 | 13 |  | 1.02 (0.58 , 1.78) | 0.956 |  | 1.03 (0.59 , 1.81) | 0.913 |
| <32 weeks | 393 | 6900 | 2 |  | 0.65 (0.16 , 2.60) | 0.539 |  | 0.66 (0.16 , 2.67) | 0.559 |
| **Cerebrovascular disease** |  |  |  |  |  |  |  |  |  |
| Term | 21175 | 368066 | 763 |  | Ref. |  |  | Ref. |  |
| 32-36 weeks | 1716 | 28495 | 58 |  | 1.13 (0.86 , 1.47) | 0.375 |  | 1.13 (0.87 , 1.48) | 0.361 |
| <32 weeks | 393 | 6779 | 17 |  | 1.40 (0.86 , 2.26) | 0.173 |  | 1.46 (0.90 , 2.38) | 0.123 |
| ^1^Adjusted for age. ^2^Adjusted for age, highest obtained educational level, parity before age 40, maternal birth year, maternal height and family history of coronary heart disease. CI = confidence interval; CVD = cardiovascular disease | | | | | | | | | |

| **Supplementary Table S18. Hazard ratios for cardiovascular events by history of preterm birth whose first birth was recorded in the MBRN.** | | | | | | | | | |
| --- | --- | --- | --- | --- | --- | --- | --- | --- | --- |
|  |  |  |  |  | Model 1**^1^** | |  | Model 2^2^ | |
|  | No. of women | Person-years | No. of events |  | Hazard ratio (95% CI) | p-value |  | Hazard ratio (95% CI) | p-value |
| **Any CVD** |  |  |  |  |  |  |  |  |  |
| Term | 17250 | 272457 | 724 |  | Ref. |  |  | Ref. |  |
| Preterm (<37 weeks) | 1855 | 29128 | 97 |  | 1.26 (1.02 , 1.56) | 0.031 |  | 1.30 (1.05 , 1.61) | 0.017 |
| **Fatal CVD** |  |  |  |  |  |  |  |  |  |
| Term | 17250 | 276813 | 54 |  | Ref. |  |  | Ref. |  |
| Preterm (<37 weeks) | 1855 | 29708 | 11 |  | 1.91 (1.00 , 3.65) | 0.051 |  | 2.20 (1.14 , 4.24) | 0.018 |
| **Myocardial infarction** |  |  |  |  |  |  |  |  |  |
| Term | 17250 | 275451 | 234 |  | Ref. |  |  | Ref. |  |
| Preterm (<37 weeks) | 1855 | 29574 | 27 |  | 1.08 (0.72 , 1.61) | 0.706 |  | 1.13 (0.75 , 1.69) | 0.557 |
| **Heart failure** |  |  |  |  |  |  |  |  |  |
| Term | 17250 | 276432 | 76 |  | Ref. |  |  | Ref. |  |
| Preterm (<37 weeks) | 1855 | 29656 | 8 |  | 0.99 (0.48 , 2.04) | 0.970 |  | 1.02 (0.49 , 2.13) | 0.953 |
| **Cerebrovascular disease** |  |  |  |  |  |  |  |  |  |
| Term | 17250 | 274323 | 389 |  | Ref. |  |  | Ref. |  |
| Preterm (<37 weeks) | 1855 | 29354 | 51 |  | 1.23 (0.92 , 1.65) | 0.158 |  | 1.25 (0.93 , 1.68) | 0.137 |
| ^1^Adjusted for age. ^2^Adjusted for age, highest obtained educational level, parity before age 40, maternal birth year, maternal height and family history of coronary heart disease. MBRN = Medical Birth Registry of Norway; CI = confidence interval; CVD = cardiovascular disease | | | | | | | | | |

| **Supplementary Table S19. Hazard ratios for cardiovascular events in women according to term or preterm first birth.** | | | | | | | | | |
| --- | --- | --- | --- | --- | --- | --- | --- | --- | --- |
|  |  |  |  |  | Model 1**^1^** | |  | Model 2^2^ | |
|  | No. of women | Person-years | No. of events |  | Hazard ratio (95% CI) | p-value |  | Hazard ratio (95% CI) | p-value |
| **Any CVD** |  |  |  |  |  |  |  |  |  |
| Term | 17435 | 267827 | 763 |  | Ref. |  |  | Ref. |  |
| Preterm (<37 weeks) | 1017 | 15160 | 53 |  | 1.22 (0.92 , 1.61) | 0.163 |  | 1.20 (0.91 , 1.59) | 0.192 |
| **Fatal CVD** |  |  |  |  |  |  |  |  |  |
| Term | 17435 | 272655 | 59 |  | Ref. |  |  | Ref. |  |
| Preterm (<37 weeks) | 1017 | 15449 | 6 |  | 1.74 (0.75 , 4.03) | 0.196 |  | 1.72 (0.74 , 3.98) | 0.207 |
| **Myocardial infarction** |  |  |  |  |  |  |  |  |  |
| Term | 17435 | 271208 | 236 |  | Ref. |  |  | Ref. |  |
| Preterm (<37 weeks) | 1017 | 15379 | 16 |  | 1.18 (0.71 , 1.97) | 0.513 |  | 1.18 (0.71 , 1.97) | 0.513 |
| **Heart failure** |  |  |  |  |  |  |  |  |  |
| Term | 17435 | 272224 | 78 |  | Ref. |  |  | Ref. |  |
| Preterm (<37 weeks) | 1017 | 15430 | 5 |  | 1.07 (0.43 , 2.66) | 0.877 |  | 1.07 (0.43 , 2.66) | 0.876 |
| **Cerebrovascular disease** |  |  |  |  |  |  |  |  |  |
| Term | 17435 | 269798 | 420 |  | Ref. |  |  | Ref. |  |
| Preterm (<37 weeks) | 1017 | 15289 | 26 |  | 1.09 (0.73 , 1.62) | 0.681 |  | 1.06 (0.72 , 1.58) | 0.756 |
| ^1^Adjusted for age. ^2^Adjusted for age, highest obtained educational level, parity before age 40, maternal birth year, maternal height and family history of coronary heart disease. CI = confidence interval; CVD = cardiovascular disease | | | | | | | | | |

| **Supplementary Table S20. Hazard ratios for cardiovascular events in women by history of preterm birth excluding women with history of stillbirth, gestational diabetes or hypertensive disorders.** | | | | | | | | | |
| --- | --- | --- | --- | --- | --- | --- | --- | --- | --- |
|  |  |  |  |  | Model 1**^1^** | |  | Model 2^2^ | |
|  | No. of women | Person-years | No. of events |  | Hazard ratio (95% CI) | p-value |  | Hazard ratio (95% CI) | p-value |
| **Any CVD** | 18975 | 327360 | 1300 |  | Ref. |  |  | Ref. |  |
| Term | 1560 | 26454 | 107 |  | 1.18 (0.97 , 1.44) | 0.101 |  | 1.21 (0.99 , 1.47) | 0.064 |
| Preterm (<37 weeks) |  |  |  |  |  |  |  |  |  |
| **Fatal CVD** | 18975 | 335812 | 163 |  | Ref. |  |  | Ref. |  |
| Term | 1560 | 27018 | 17 |  | 1.68 (1.02 , 2.78) | 0.042 |  | 1.82 (1.10 , 3.02) | 0.021 |
| Preterm (<37 weeks) |  |  |  |  |  |  |  |  |  |
| **Myocardial infarction** | 18975 | 332838 | 423 |  | Ref. |  |  | Ref. |  |
| Term | 1560 | 26840 | 32 |  | 1.09 (0.76 , 1.56) | 0.644 |  | 1.15 (0.80 , 1.65) | 0.452 |
| Preterm (<37 weeks) |  |  |  |  |  |  |  |  |  |
| **Heart failure** | 18975 | 334938 | 183 |  | Ref. |  |  | Ref. |  |
| Term | 1560 | 26993 | 9 |  | 0.79 (0.40 , 1.55) | 0.494 |  | 0.81 (0.41 , 1.60) | 0.548 |
| Preterm (<37 weeks) |  |  |  |  |  |  |  |  |  |
| **Cerebrovascular disease** | 18975 | 331379 | 683 |  | Ref. |  |  | Ref. |  |
| Term | 1560 | 26668 | 52 |  | 1.10 (0.83 , 1.46) | 0.505 |  | 1.11 (0.84 , 1.48) | 0.456 |
| Preterm (<37 weeks) | 18975 | 327360 | 1300 |  | Ref. |  |  | Ref. |  |
| ^1^Adjusted for age. ^2^Adjusted for age, highest obtained educational level, parity before age 40, maternal birth year, maternal height and family history of coronary heart disease. CI = confidence interval; CVD = cardiovascular disease | | | | | | | | | |

| **Supplementary Table S21. Hazard ratios for validated cardiovascular events in women according to preterm birth history.** | | | | | | | | | |
| --- | --- | --- | --- | --- | --- | --- | --- | --- | --- |
|  |  |  |  |  | Model 1**^1^** | |  | Model 2^2^ | |
|  | No. of women | Person-years | No. of events |  | Hazard ratio (95% CI) | p-value |  | Hazard ratio (95% CI) | p-value |
| **Myocardial infarction** |  |  |  |  |  |  |  |  |  |
| Term | 21175 | 369670 | 486 |  | Ref. |  |  | Ref. |  |
| Preterm (<37 weeks) | 2109 | 35528 | 47 |  | 1.15 (0.85 , 1.55) | 0.362 |  | 1.20 (0.89 , 1.62) | 0.239 |
| **Heart failure** |  |  |  |  |  |  |  |  |  |
| Term | 21175 | 372086 | 207 |  | Ref. |  |  | Ref. |  |
| Preterm (<37 weeks) | 2109 | 35720 | 15 |  | 0.96 (0.57 , 1.62) | 0.868 |  | 0.97 (0.57 , 1.65) | 0.907 |
| **Cerebrovascular disease** |  |  |  |  |  |  |  |  |  |
| Term | 21175 | 368066 | 746 |  | Ref. |  |  | Ref. |  |
| Preterm (<37 weeks) | 2109 | 35274 | 71 |  | 1.14 (0.90 , 1.46) | 0.280 |  | 1.16 (0.90 , 1.48) | 0.244 |
| ^1^Adjusted for age. ^2^Adjusted for age, highest obtained educational level, parity before age 40, maternal birth year, maternal height and family history of coronary heart disease. CI = confidence interval; CVD = cardiovascular disease | | | | | | | | | |

| **Supplmentary Table 22. Hazard ratios for cardiovascular events in women with history of preterm birth and without history of LGA or SGA offspring.** | | | | | | | | | |
| --- | --- | --- | --- | --- | --- | --- | --- | --- | --- |
|  |  |  |  |  | Model 1**^1^** | |  | Model 2**^2^** | |
|  | No. of women | Person-years | No. of events |  | Hazard ratio | p-value |  | Hazard ratio | p-value |
| **Any CVD** |  |  |  |  |  |  |  |  |  |
| Term | 14066 | 242570 | 996 |  | Ref. |  |  | Ref. |  |
| Preterm (<37 weeks) | 1043 | 17602 | 72 |  | 1.13 (0.89 , 1.43) | 0.324 |  | 1.16 (0.91 , 1.47) | 0.233 |
| **Fatal CVD** |  |  |  |  |  |  |  |  |  |
| Term | 14066 | 249072 | 124 |  | Ref. |  |  | Ref. |  |
| Preterm (<37 weeks) | 1043 | 18014 | 11 |  | 1.52 (0.82 , 2.81) | 0.187 |  | 1.66 (0.89 , 3.08) | 0.111 |
| **Myocardial infarction** |  |  |  |  |  |  |  |  |  |
| Term | 14066 | 246818 | 325 |  | Ref. |  |  | Ref. |  |
| Preterm (<37 weeks) | 1043 | 17879 | 23 |  | 1.10 (0.72 , 1.68) | 0.667 |  | 1.17 (0.76 , 1.79) | 0.475 |
| **Heart failure** |  |  |  |  |  |  |  |  |  |
| Term | 14066 | 248432 | 136 |  | Ref. |  |  | Ref. |  |
| Preterm (<37 weeks) | 1043 | 17969 | 8 |  | 1.00 (0.49 , 2.04) | 0.998 |  | 1.04 (0.51 , 2.14) | 0.906 |
| **Cerebrovascular disease** |  |  |  |  |  |  |  |  |  |
| Term | 14066 | 245577 | 524 |  | Ref. |  |  | Ref. |  |
| Preterm (<37 weeks) | 1043 | 17786 | 34 |  | 1.02 (0.72 , 1.44) | 0.927 |  | 1.03 (0.72 , 1.46) | 0.879 |
| ^1^Adjusted for age. ^2^Adjusted for age, highest obtained educational level, parity before age 40, maternal birth year, maternal height and family history of coronary heart disease. SGA = small for gestational age; LGA = large for gestational age; CI = confidence interval; CVD = cardiovascular disease | | | | | | | | | |

| **Supplementary Table S23. Decomposition of the association between history of preterm birth and cardiovascular disease into direct and indirect effects among women who had their cardiovascular risk factors measured or collected after age 40.** | | | | | | | | | | | | |
| --- | --- | --- | --- | --- | --- | --- | --- | --- | --- | --- | --- | --- |
|  |  |  | Total effect^1^ | |  | Direct effect^2^ | |  | Indirect effect^3^ | |  | Proportion explained^4^ |
| **Cardiovascular risk factor** | No. of women |  | HR (95% CI) | p-value |  | HR (95% CI) | p-value |  | HR (95% CI) | p-value |  | Percentage |
| BMI^5^ | 17576 |  | 1.14 (0.93 , 1.40) | 0.213 |  | 1.20 (0.96 , 1.49) | 0.117 |  | 0.95 (0.88 , 1.04) | 0.267 |  | -36 |
| Systolic blood pressure^5^ | 17568 |  | 1.13 (0.93 , 1.39) | 0.223 |  | 1.17 (0.93 , 1.46) | 0.174 |  | 0.97 (0.89 , 1.06) | 0.529 |  | -23 |
| Diastolic blood pressure^5^ | 17570 |  | 1.13 (0.94 , 1.37) | 0.202 |  | 1.16 (0.95 , 1.42) | 0.153 |  | 0.98 (0.89 , 1.07) | 0.594 |  | -19 |
| Serum glucose^5^ | 17191 |  | 1.14 (0.93 , 1.39) | 0.196 |  | 1.17 (0.95 , 1.44) | 0.149 |  | 0.98 (0.90 , 1.07) | 0.594 |  | -18 |
| Serum non-HDL cholesterol^5^ | 16854 |  | 1.14 (0.92 , 1.40) | 0.222 |  | 1.18 (0.94 , 1.48) | 0.145 |  | 0.96 (0.88 , 1.06) | 0.436 |  | -28 |
| Smoking^6^ | 17455 |  | 1.23 (1.02 , 1.49) | 0.033 |  | 1.22 (1.00 , 1.50) | 0.054 |  | 1.01 (0.93 , 1.09) | 0.898 |  | 2 |
| \| ^1^Association between history of preterm birth and CVD compared to women with history of term birth. ^2^Part of the association between history of preterm birth and CVD that is not explained by the cardiovascular risk factor. ^3^Part of the association between history of preterm birth and CVD that is explained by the cardiovascular risk factor.  ^4^Proportion excess cardiovascular risk in women with history of preterm birth that is explained by cardiovascular risk factor. ^5^Estimates are adjusted for age (used as time scale  in the Cox proportional hazards model), age at measurement of the cardiovascular risk factor, highest obtained educational level, age at first birth, parity, first birth in the  Medical Birth Registry of Norway, maternal height and birth year. ^6^Estimates are adjusted for age (used as time scale in the Cox proportional hazards model), highest obtained educational level, age at first birth, parity, first birth in the Medical Birth Registry of Norway, maternal height and birth year. CVD = cardiovascular disease;  CI = confidence interval, BMI = body mass index; HDL = high-density lipoprotein \| \| --- \| | | | | | | | | | | | | |

| **Supplementary Table S24. Decomposition of the association between history of preterm birth and cardiovascular disease into direct and indirect effects where cardiovascular risk factors were measured at women’s first HUNT exam.** | | | | | | | | | | | | |
| --- | --- | --- | --- | --- | --- | --- | --- | --- | --- | --- | --- | --- |
|  |  |  | Total effect^1^ | |  | Direct effect^2^ | |  | Indirect effect^3^ | |  | Proportion explained^4^ |
| **Cardiovascular risk factor** | No. of women |  | HR (95% CI) | p-value |  | HR (95% CI) | p-value |  | HR (95% CI) | p-value |  | Percentage |
| BMI^5^ | 22862 |  | 1.25 (1.05 , 1.49) | 0.011 |  | 1.27 (1.06 , 1.53) | 0.011 |  | 0.98 (0.92 , 1.06) | 0.686 |  | -7 |
| Systolic blood pressure^5^ | 22865 |  | 1.25 (1.05 , 1.49) | 0.012 |  | 1.25 (1.04 , 1.51) | 0.016 |  | 1.00 (0.93 , 1.07) | 0.932 |  | -1 |
| Diastolic blood pressure^5^ | 22861 |  | 1.25 (1.05 , 1.49) | 0.012 |  | 1.24 (1.03 , 1.49) | 0.026 |  | 1.01 (0.94 , 1.09) | 0.778 |  | 5 |
| Serum glucose^5^ | 20952 |  | 1.24 (1.03 , 1.49) | 0.024 |  | 1.25 (1.02 , 1.54) | 0.030 |  | 0.99 (0.91 , 1.07) | 0.736 |  | -6 |
| Serum non-HDL cholesterol^5^ | 20521 |  | 1.29 (1.06 , 1.56) | 0.012 |  | 1.31 (1.06 , 1.62) | 0.011 |  | 0.98 (0.90 , 1.06) | 0.593 |  | -9 |
| Smoking^6^ | 20628 |  | 1.28 (1.06 , 1.54) | 0.010 |  | 1.28 (1.05 , 1.57) | 0.015 |  | 1.00 (0.92 , 1.08) | 0.953 |  | -1 |
| ^1^Association between history of preterm birth and CVD compared to women with history of term birth. ^2^Part of the association between history of preterm birth and CVD that is not explained by the cardiovascular risk factor. ^3^Part of the association between history of preterm birth and CVD that is explained by the cardiovascular risk factor. ^4^Proportion excess cardiovascular risk in women with history of preterm birth that is explained by cardiovascular risk factor. ^5^Estimates are adjusted for age (used as time scale in the Cox proportional hazards model), age at measurement of the cardiovascular risk factor, highest obtained educational level, age at first birth, parity, first birth in the Medical Birth Registry of Norway, maternal height and birth year. ^6^Estimates are adjusted for age (used as time scale in the Cox proportional hazards model), highest obtained educational level, age at first birth, parity, first birth in the Medical Birth Registry of Norway, maternal height and birth year. CVD = cardiovascular disease;  CI = confidence interval, BMI = body mass index; HDL = high-density lipoprotein | | | | | | | | | | | | |
|  | | | | | | | | | | | | |
